# Ethanolic extract of *Morinda citrifolia* improves gut microbiota, intestinal morphology, and performance without adverse effects on hematological profiles in broiler chickens

**DOI:** 10.3389/fvets.2025.1686136

**Published:** 2026-01-28

**Authors:** Daniel Marco Paredes-López, Rizal Robles-Huaynate, Rosa Amelia Perales-Camacho, Cindy Vanessa Alania-Santiago, Uriel Aldava-Pardave

**Affiliations:** 1 Department of Animal Science, Universidad Nacional Agraria de la Selva, Tingo María, Peru; 2 Department of Animal Health and Public Health, Faculty of Veterinary Medicine, Universidad Nacional Mayor de San Marcos, Lima, Peru; 3 Posgraduate School, Universidad Nacional Agraria de la Selva, Tingo María, Peru; 4 Posgraduate School, Universidad Nacional Agraria La Molina, Lima, Peru

**Keywords:** ethanolic extract, gut health, gut histology, microbiota, *Morinda citrifolia*, growth performance

## Abstract

Over the last six decades, the extensive use of antibiotics as growth promoters in animal nutrition has contributed to antimicrobial resistance and ultimately poses a risk to public and environmental health. Therefore, there is a growing interest in alternative strategies, such as plant extracts and essential oils, to address this issue. The aim of this study was to evaluate the effect of *Morinda citrifolia* leaf ethanolic extract (MCEE) on the intestinal health and performance of broiler chickens. A total of 360 Cobb 500 broilers were randomly distributed into groups C1, C2, S1, S2, and S3, with six replicates of 12 chickens each. C1 was fed the base diet (BD), and C2 was fed BD + 50 ppm zinc bacitracin (ZB). S1, S2, and S3 were supplemented with MCEE in daily doses of 5.62, 11.0, and 16.3 mg/kg BW, respectively, from 1 to 21 days of age. Hematological profiles and gut histology were assessed at 7, 14, and 21 days of age, while the abundance of microbiota in the ileum was measured at 21 and 28 days of age. Performance indices, including weight gain, feed intake, and feed conversion rate, were evaluated at 7, 21, and 33 days of age. Data were analyzed using a general factorial arrangement. The abundance of *Escherichia coli* and *Staphylococcus aureus* log (CFU/g) decreased in the three supplemented groups by 21 days of age, and these levels were maintained until 28 days of age. Additionally, in the three supplemented groups, the abundance of *Lactobacillus* sp. decreased by 21 days of age; however, it was re-established by 28 days of age. Villus height and crypt depth increased with age in the S2 group by 21 days of age. Performance indices improved during the fattening phase overall in chickens in group S2. In conclusion, a daily dose of 11.0 mg/kg BW MCEE in the diet improved performance indices by modulating gut microbiota and histology at 21 days in broiler chickens.

## Introduction

The overuse of antibiotics, commonly employed to reduce pathogens in the gut, control gut infections, and promote the growth of livestock and poultry, may contribute to antimicrobial resistance. Additionally, animal-derived food products can contain antibiotic residues, thereby compromising public and environmental health (72).

This scenario prompts the exploration of new methods for controlling these pathogens through alternative sources, such as plant extracts or essential oils that contain active antimicrobial, antioxidant, and immunomodulatory compounds, which collectively modulate gut microbiota (1), enhance gut health (2, 3) in livestock and poultry. This promotes the improvements in wellbeing, health (4), and performance (5) of these animal species.

*Morinda citrifolia*, “noni,” is a plant native to Southeast Asia that has expanded throughout the Caribbean and Central, North, and South America (6). It is known for its antimicrobial properties (2) and its immunomodulatory activities (7). This antimicrobial activity has been attributed to noni fruit phenolics and polysaccharide extracts, which ameliorate gut microbiota dysbiosis by increasing short-chain fatty acid-producing bacteria and decreasing lipopolysaccharide-producing bacteria, resulting in an improvement in gut mucosa structure and a reduction in gut inflammation (8, 9). Furthermore, previous studies have reported improvements in the productive indices of tilapia and guinea pigs using fruit extract and fruit powder (10, 11). Despite its traditional medicinal and nutraceutical use in the Peruvian Amazon and its year-round production of abundant foliage and fruits, the plant’s productive potential remains underexploited. Polyphenols, flavonoids, tannins, triterpenes, and steroids are the main components of noni leaf extract (12, 13), and improvements in chicken performance indices have been reported (2). Noni leaf extracts differ in very few phytocompounds from those obtained in noni fruit extracts, in which triterpenes and steroids have not been reported (67), and no variation in chicken performance indices has been observed with noni fruit powder and extract (14, 15). The aim of this study was to evaluate the effect of *Morinda citrifolia* leaf ethanolic extract (MCEE) on gut health and growth performance as an alternative to antibiotics for growth promotion in broiler chickens.

## Materials and methods

### Preparation of the extract and chemical characterization

Leaves of noni were collected from trees in the Luyando district of the Leoncio Prado province in the Huánuco region of Peru, located at 9°14′49″ south latitude and 75°59′31″ west longitude, and at an elevation of 620 m.a.s.l. During the morning hours, 300 g of fully developed intermediate leaves were collected per tree from 10 randomly selected trees, as this stage contains the highest quantity of accumulated phytocompounds, ensuring homogeneity in color and size. The leaves were carefully cleaned with distilled water and dried in an oven (Memmert, UN110 Plus, Germany) at 40 °C for 72 h. Next, the leaves were ground in a Model 4 Thomas Wiley brand grinder (USA) until a thick powder was obtained and then passed through a set of 1 mm diameter sieves to homogenize the particle size. The extract was obtained using an ethanolic extraction method followed by phytochemical screening. For this purpose, 50 g of powdered and sieved noni leaf was placed on filter paper to form the cartridge, which was then inserted into the Soxhlet apparatus. Next, 150 mL of ethanol at 70% concentration was placed in an Erlenmeyer flask in a proportion of 1:3 (w/v) to extract the ethanol-soluble metabolites for 2 h. Subsequently, the ethanol extract was filtered through Whatman No. 40 filter paper, after which the ethanolic extract was dehydrated in a rotary evaporator (Heidolp, Germany) at 40 °C and reduced pressure to eliminate all the solvent and achieve complete dryness (16).

Once the ethanolic extract was obtained, phytochemical screening was carried out to identify the phytochemical groups such as alkaloids, oils, triterpenes and steroids, phenolic compounds, quinones, flavonoids, saponins, and tannins through chemical color and precipitation tests, following the method described by Ajayi et al. (17). In addition, chromatographic profiles using high-performance liquid chromatography (HPLC-MS) were employed to determine the levels of polyphenols and total flavonoids in the ethanol extract of *Morinda citrifolia*. For this purpose, a column R-18, a flow rate of 1 mL/min, an injection volume of 10 μL, a mobile phase of toluene, ethyl acetate, and formic acid (6:4:0.3 mL, v/v), and a wavelength of 254 nm were used.

### Treatments, animals, and experimental management

The study was conducted at the Faculty of Zootechnics of the Universidad Nacional Agraria de la Selva, located in the province of Leoncio Prado, Department of Huánuco, Peru (09°17′58″ south latitude and 76°01′07″ west longitude), at an elevation of 660 m a.s.l. The site’s climate is classified as humid tropical, characterized by an annual precipitation of 3,293 mm, an average annual temperature of 24.85 °C, and a relative humidity of 80% (18). During the experimental period, wood shaving bedding, with 18 h of lighting and manual ventilation, and the daily average temperature and humidity in the chicken houses ranged from 28 to 31 °C and from 30 to 70%, respectively. The evaluated supplementations were as follows:

- Control Group 1 (C1): basal diet
- Control Group 2 (C2): basal diet + 50 ppm zinc bacitracin (BZ)
- Supplementation 1 (S1): basal diet + 5.62 mg/kg body weight MCE
- Supplementation 2 (S2): basal diet + 11.0 mg/kg body weight MCE
- Supplementation 3 (S3): basal diet + 16.3 mg/kg body weight MCEE

In total, 360 one-day-old male Cobb 500 chicks (40 ± 0.43 g body weight) were used, evenly distributed among the five supplementations, with six experimental units per treatment, each consisting of 12 chicks housed in the same pen. The 12 chicks in each experimental unit were group-housed in a 1 m^2^ pen from day 1 to day 33, and their development was divided into the following stages: starter (1–7 days), grower (8–21 days), and finisher (22–33 days). The diet was formulated using the Mixit-2 program according to Rostagno et al. (19), and a micronutrient premix was prepared with insoluble crude fiber to ensure good homogenization, followed by final mixing of all ingredients in a 100 kg-capacity horizontal mixer for 10 min. The diet was offered ad libitum in powdered form in 3 kg-capacity plastic cylindrical feeders at an average daily dose per chick of 26.3 g, 76.85 g, and 144.43 g for the starter, grower, and finisher stages, respectively. For the *Morinda citrifolia* ethanolic extract (MCEE), its intake was ensured by administering it in one-third of the chickens’ daily drinking water in 2 L cylindrical drinkers from day 1 to day 21, using a concentrated solution of 100 mg MCEE/mL in a 0.9% sterile sodium chloride solution, which was stored throughout the experimental period at 4 °C in 2 mL Eppendorf tubes protected from light with aluminum foil. The average water intake per chick was 65.75 mL, 192.5 mL, and 361.10 mL for the starter, grower, and finisher stages, respectively. The formulated diets and chemical composition of the basal diet used in each chicken stage (starter, grower, and finisher) are shown in Tables 1, 2. Zinc Bacitracin was added to the diet of C2 as a growth-promoting antibiotic to serve as a positive control for contrasting the effect of MCEE as a growth promoter.

**Table 1:** Experimental diets formulated for the male broiler chickens during the initial (1 to 7 days of age), growth (8 to 21 d of age) and fattening (22 to 33 d of age) stages.

Ingredients	Initial			Growth			Fattening		
	C1	C2	S1-S3	C1	C2	S1-S3	C1	C2	S1-S3
Corn	51.1	51.2	51.3	51.2	51.2	51.2	53.96	53.96	53.96
Palm oil	4.46	4.46	4.46	4.46	4.46	4.46	5.5	5.5	5.5
Soybean cake (46%)	39.7	39.9	39.7	39.9	39.9	39.88	36.37	36.37	36.37
Calcium carbonate	0.89	0.79	0.75	0.79	0.79	0.79	0.75	0.75	0.75
Dicalcium phosphate	1.8	1.8	1.8	1.8	1.8	1.8	1.58	1.58	1.58
Salt	0.23	0.22	0.25	0.22	0.22	0.22	0.2	0.2	0.2
Vit + Min Pre-Mix	0.15	0.15	0.15	0.15	0.15	0.15	0.1	0.1	0.1
Aflaban	0.05	0.05	0.05	0.05	0.05	0.05	0.05	0.05	0.05
Coccidiostat	0.05	0.05	0.05	0.05	0.05	0.05	0.05	0.05	0.05
Butylated Hydroxytoluene	0.05	0.05	0.05	0.05	0.05	0.05	0.05	0.05	0.05
Choline chloride	0.25	0.2	0.21	0.2	0.2	0.2	0.2	0.2	0.2
Sodium butyrate	0.1	0.1	0.11	0.1	0.1	0.1	0.1	0.1	0.1
Sodium bicarbonate	0.46	0.45	0.44	0.45	0.45	0.45	0.44	0.44	0.44
Lysine (78.4%)	0.31	0.22	0.24	0.22	0.22	0.22	0.24	0.24	0.24
Methionine (99%)	0.25	0.23	0.28	0.23	0.23	0.23	0.22	0.22	0.22
Threonine (98%)	0.11	0.09	0.09	0.09	0.09	0.09	0.09	0.09	0.09
Valine (99%)	0.09	0.06	0.06	0.06	0.06	0.06	0.06	0.06	0.06
BMD (10%)	0	0.01	0	0	0.01	0	0	0.05*	0
Oxytetracycline (99%)	0	0	0	0	0	0	0	0	0
Total	100	100	100	100	100	100	100	100	100

C1: Base diet; C2: Base diet+Zinc bacitracin; S1-S3: Supplemented with *M. citrifolia* ethanolic extract; *Supplemented up to 21 days of age.

**Table 2:** Proximal analysis of the experimental diets for male broiler chickens during the initial, growth and finishing stages (1 to 33 d of age).

Diet	Components (%)						
	Supplement	Dry matter	Ash	Raw protein	Ethereal extract	Total fiber	ELN
Initial base	C1, S1, S2, S3	90.10	7.12	23.5	5.23	2.43	52. 27
Initial with ZB	C2	90.05	7.10	23.15	5.19	2.49	52.12
Growth base	C1, S1, S2, S3	91.24	6.81	22.13	7.02	2.32	52.96
Growth with ZB	C2	91.52	6.86	22.04	7.16	2.35	53.11
Fattening base	C1, S1, S2, S3	88.72	6.12	20.34	7.92	2.40	51.91
Fattening with ZB	C2	89.05	6.11	20.41	7.91	2.46	52.14

ZB: zinc bacitracin at 50 ppm in the die, S1: 5.62 mg/Kg BW, S2: 11.0 mg/Kg BW, S3: 16.3 mg/Kg BW of MCEE.

### Blood samples and hematological profile

Blood samples were collected via jugular vein puncture from one randomly selected chick per experimental unit in each of the five treatments on days 7, 14, and 21 of age. A total of 30 samples were obtained each sampling day and placed in 2 mL Vacutainer tubes containing 2 mg of high molecular weight heparin. Blood samples were analyzed immediately after collection and kept refrigerated at 6 °C until laboratory procedures were completed. Hematocrit, hemoglobin, total erythrocyte, and leukocyte quantification were determined. The erythrocyte count, total and differential leukocyte counts, hematocrit, and hemoglobin levels were determined using the Neubauer chamber, Wright–Giemsa-stained slides, microhematocrit capillaries, and cyanmethemoglobin methods, respectively. Microhematocrit capillaries were centrifuged at 11,000 rpm for 10 min in a Kert Lab Tom’s (USA Science Tech Group) centrifuge, and hemoglobin profiles were determined using an Auto Chemistry Analyzer-AS 830 spectrophotometer (Italy) at 515 and 530 nm, with a specific kit (QAC-Espain). At the same time, the indices for mean corpuscular volume (MCV), mean corpuscular hemoglobin (MCH), and mean corpuscular hemoglobin concentration (MCHC) were calculated using standardized formulas based on hematocrit, erythrocyte counts, and hemoglobin profiles (20).

### Intestine content and microbial culture

Three chicks from three different experimental units (replicates) were randomly selected from each treatment at 21 and 28 days of age, resulting in a total of 15 chicks per sampling day, which were euthanized by atlanto-occipital dislocation. The digestive tracts were immediately dissected, and a segment of approximately 30 cm was taken from the ileum after Meckel’s diverticulum (21, 22). It was opened to obtain 1 g of intestinal content, including scraping the mucus and collecting it in sterile Petri dishes. Bacteria from the native microbial species in the broiler chicken microbiota, such as *Escherichia coli*, *Lactobacillus* sp., and *Staphylococcus* sp., were determined (21). MacConkey, MRS, and mannitol salt agar were used for *E. coli*, *Lactobacillus* sp., and *Staphylococcus aureus* growth, respectively (Merck, Darmstadt, Germany). One gram of the sample was suspended in 9 mL of 0.9% NaCl sterile diluent, stirred by a mixer device, and then 10-fold dilutions were performed up to 10^−5^ for each sample. Then, 1 mL of each dilution was mixed with 10 mL of the selective culture media for each of the three bacteria in sterile Petri dishes in duplicate series, and after 10 min, the surface was covered with 10 mL of agar to prevent contamination. Finally, Petri dishes for *S. aureus* and *E. coli* were incubated in aerobic conditions, while those for *Lactobacillus* sp. were incubated in a microaerophilic environment (4% O₂ and 5–10% CO₂) for 24 h at 37 °C. Identification of colonies for *S. aureus*, *E. coli*, and *Lactobacillus* sp. was done by the color of the colonies resulting from the biochemical reactions of each bacterium in its selective culture media. The bacterial count was expressed as the natural logarithm of the number of colony-forming units per gram (log CFU/g) of ileal content (21, 22).

### Gut histology

Six chicks from six different experimental units (replicates) were randomly selected from each treatment at 7, 14, and 21 days of age, resulting in a total of 30 chicks per sampling day, which were euthanized by atlanto-occipital dislocation (23). Their gut tracts were immediately dissected, and a 5 cm segment from the middle part of the duodenum, jejunum, and ileum (21, 22) was opened lengthwise to create transverse sections. These segments were removed and submerged three to four times in sterile 9 g/L sodium chloride solution to detach the gut content from the mucosa, placed with staples on a thick cardboard base to keep the segments straight, and three segments from each bird were set in 100 mL of 10% formalin in a 9 g/L sodium chloride solution (1/10 W/V). Fixed samples from the intestines were processed using conventional histological methods and stained with hematoxylin and eosin (24).

**Figure 1:**
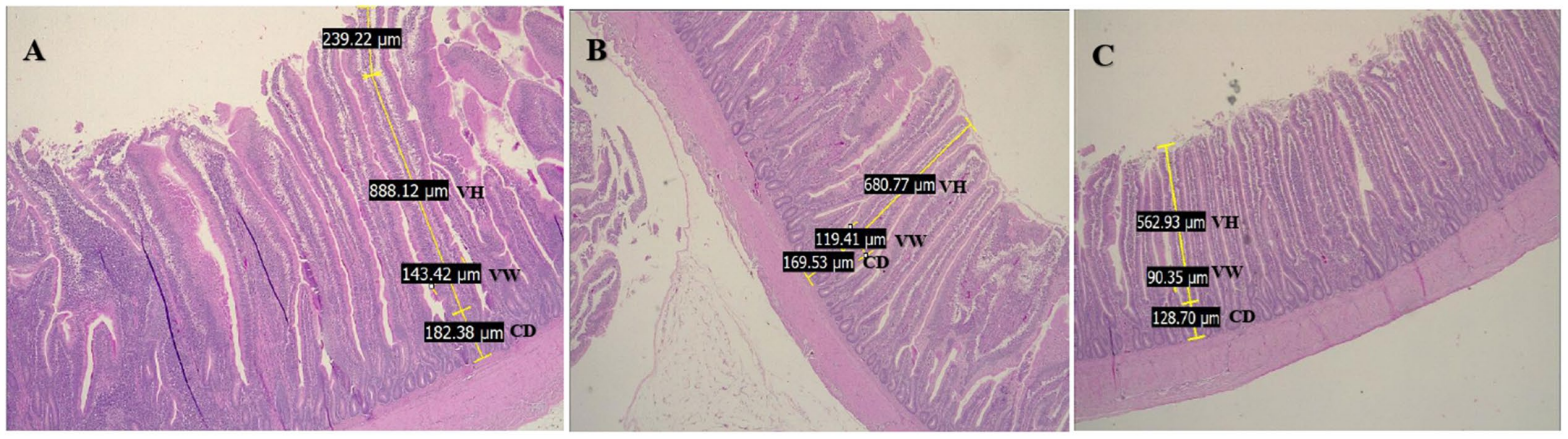
Villous height (VH), villous width (VW), and crypt depth (CD) measures in the duodenum **(A)**, jejunum **(B)**, and ileum **(C)** of broiler chicken supplemented *Morinda citrifolia* ethanolic extract (10×, hematoxylin and eosin staining).

Tissue slides were examined using a blind reading procedure, as the pathologist had no knowledge of the treatment group of the experimental chickens during the histological evaluation. Villi for measurement were selected at random from a microscope field; the height and width of the gut villi and the depth of the crypts were measured in ten villi at 10x magnification, due to the well-developed nature of these structures in the chicken gut. A Leica DM500 optical microscope was used, and the LAS EZ Leica program was installed on a computer connected to the Leica microscope, which included a Leica ICC50 camera. The system allows users to measure the distance between two fixed points. The measurements were recorded in microns (μm) (Figure 1), and the average measurements for each gut segment in each bird were noted.

### Growth performance

Weight gain, feed intake, and feed conversion rate are the primary indices for evaluating animal production performance (25). All chickens in the six replicates of the five experimental groups, as well as the feed administered to these chickens, were weighed at 7, 21, and 33 days of age after withdrawing feed for 12 h prior to weighing. This was done using a Xing Yun balance, Model XY3KB1EB, 3 Kg/0.1 g (China). Total feed intake (TFI) was calculated based on the total feed consumption of the lot and the number of birds in each supplemented group over the specified period. Weight gain (WG) was calculated as the difference between final weight and initial weight of the lot divided by the number of birds in each supplemented group. Feed conversion rate (FCR) was calculated based on the total feed consumption of the lot and the final weight gain of the lot in each supplemented group. Chicken mortality was recorded at 1.10% (4 chickens) of the lot. All performance indices were measured per pen.

### Statistical analysis

A completely randomized design was used, in which the experimental units were represented by pens containing 12 chickens, and each treatment consisted of six experimental units or replicates. Body weight was recorded individually and averaged per replicate to ensure data independence, while the other variables were assessed in a randomly selected chicken per replicate. Statistical analyses were performed using R software version 4.4.0 (Supplementary material Table 5) (26) via the RStudio interface (27). Microbiological variables were analyzed using two-way ANOVA (age and treatment), with negative binomial models due to the nature of the count data. Gut histology was assessed using a three-way ANOVA (intestinal segment, age, and treatment), utilizing lognormal or Gamma distribution models depending on the variable analyzed. Hematological parameters were analyzed using a two-way ANOVA (age and treatment) with a non-parametric approach based on transformed aligned ranks. Growth performance was analyzed using one-way ANOVA (treatment), assuming a normal distribution; when the parametric assumptions were not met, the non-parametric Kruskal–Wallis test was used. Multiple comparisons were performed at a significance level of 0.05 using the false discovery rate (FDR) correction, which controls the expected proportion of false positives while maintaining adequate statistical power (28).

## Results

Phytochemical screening of the *Morinda citrifolia* ethanolic extract (MCEE) showed low reactions to flavonoids, phenolic compounds, triterpenes and steroids, sugar reducers, quinones, and amino acids (Table 3). In the present study, 1.5 mg of GAE/g of dry ethanolic extract for the phenolic compounds and 0.17 mg of QE/g of dry extract for flavonoids were obtained on a dry basis. In addition, MCEE inhibited 58% of the DPPH radicals with a CI50 of 137.89 μg/mL.

**Table 3:** Phytochemical screening of the dichloromethane, ethanol and aqueous extract from the *Morinda citrifolia* leaves.

Metabolites	Trial	Solvent		
		D	E	W
Oils and fats	Sudan III	+	ND	ND
Alkaloids	Dragendorff	−	−	−
	Mayer	−	−	−
	Wagner	−	−	−
Phenol compounds	Iron (III) Chloride	ND	*+*	ND
Flavonoids	Shinoda	ND	*+*	+
Anthocyanidins	Anthocyanidin	ND	*−*	ND
Catechins	Catechins	ND	*−*	ND
Tannins	Gelatin	ND	ND	+
Lactones and coumarins	Baljet	+	−	ND
Triterpenes and steroids	Lieberman–Buchard	++	+	ND
Cardenolides	Kedde	ND	*−*	ND
Quinones	Bornträger	ND	*+*	ND
Saponins	Foam	ND	*−*	*−*
Resins	Resins	ND	−	ND
Sugar reducers	Felhing	ND	+	+
Mucilage	Mucilage	ND	ND	−
Amino acids	Ninhydrin	ND	+	ND
Bitter and astringent substances	Bitter and Astringent Substances	ND	ND	+

D, dichloromethane; E, ethanol; W, water; ND, undetermined; +, Low; −, No reaction.

### Hematological profiles

Hematocrit, hemoglobin, erythrocyte, leukocyte, lymphocyte, and granulocyte profiles are presented in Table 4. The HB, MCH, and MCHC profiles of the chickens supplemented with 16.3 mg/Kg BW MCEE increased at 21 days of age compared to the chickens from the C1 group (*p* = 0.01, *p* = 0.04, and *p* = 0.02, respectively). Nonetheless, the hematocrit, erythrocyte, leukocyte, lymphocyte, granulocyte profiles, and MCV index did not change in chickens in S1, S2, or S3 (*p* > 0.05) (Table 4).

**Table 4:** ANOVA and blood profiles of broiler chickens supplemented with different levels of *Morinda citrifolia* ethanolic extract (Mean ± SD) (*n* = 30).

Factors	Blood profiles and indices								
	HTO (%)	HB (gdL^−1^)	*ERY (x10^6^mm-^3^)	*LEU (x10^3^mm-^3^)	LYN (%)	GRA (%)	MCV (fL)	MCH (pg)	MCHC (gdL^−1^)
Age (days)									
7	26.27 ± 2.02	19.00 ± 0.33	15.11 ± 0.05	8.67 ± 0.23c	47.03 ± 8.58	53.63 ± 8.05	71.95 ± 1.93ab	51.53 ± 5.57	71.75 ± 8.67
14	26.27 ± 1.20	19.99 ± 1.30	15.12 ± 0.04	9.32 ± 0.20b	43.83 ± 3.65	56.27 ± 3.63	71.43 ± 1.36b	54.40 ± 3.32	76.18 ± 4.79
21	28.47 ± 6.52	19.58 ± 2.20	15.16 ± 0.16	9.52 ± 0.14a	43.60 ± 4.38	54.97 ± 4.19	73.37 ± 4.06a	52.61 ± 10.63	72.47 ± 17.27
Supplementation									
C1	27.06 ± 6.12	18.95 ± 1.06	15.13 ± 0.15	9.29 ± 0.47	43.22 ± 4.40	56.06 ± 4.35	72.15 ± 3.80	51.39 ± 7.26	71.77 ± 12.51
C2	27.28 ± 4.57	19.34 ± 1.49	15.14 ± 0.11	9.19 ± 0.42	44.94 ± 4.89	55.00 ± 3.82	72.55 ± 2.80	52.14 ± 7.17	72.21 ± 11.55
S1	27.67 ± 4.30	20.01 ± 1.72	15.15 ± 0.10	9.21 ± 0.51	46.33 ± 9.04	53.39 ± 8.05	72.61 ± 3.14	53.27 ± 6.74	73.75 ± 11.32
S2	26.56 ± 2.31	19.12 ± 1.07	15.12 ± 0.07	9.24 ± 0.40	44.61 ± 5.90	55.61 ± 5.81	72.21 ± 2.06	52.64 ± 4.59	73.09 ± 8.12
S3	26.44 ± 2.09	20.21 ± 1.85	15.12 ± 0.05	9.22 ± 0.28	45.00 ± 5.36	54.72 ± 5.73	71.72 ± 2.04	54.80 ± 9.71	76.53 ± 14.04

Supplementation by age		Blood profiles and indices								
Supplementation	Age (days)	HTO (%)	HB (gdL^−1^)	*ERY (x10^6^mm-^3^)	*LEU (x10^3^mm-^3^)	LYN (%)	GRA (%)	MCV (fL)	MCH (pg)	MCHC (gdL^−1^)
C1	7	24.67 ± 1.21	18.62 ± 0.63	15.07 ± 0.03	8.72 ± 0.34	46.00 ± 6.16ab	54.00 ± 6.16	70.12 ± 1.66	51.32 ± 2.63	73.23 ± 4.31
C2	7	25.67 ± 2.34	19.10 ± 0.00	15.09 ± 0.06	8.60 ± 0.17	47.17 ± 5.34ab	54.50 ± 3.15	71.53 ± 2.17	53.18 ± 6.16	74.56 ± 10.24
S1	7	26.67 ± 1.37	19.10 ± 0.00	15.12 ± 0.03	8.47 ± 0.14	54.00 ± 12.60a	47.67 ± 12.31	72.37 ± 1.63	52.02 ± 3.47	72.00 ± 6.34
S2	7	26.50 ± 1.52	19.10 ± 0.00	15.11 ± 0.04	8.67 ± 0.11	46.33 ± 8.80ab	53.67 ± 5.80	72.56 ± 1.12	53.53 ± 3.91	73.83 ± 6.24
S3	7	27.83 ± 2.40	19.10 ± 0.00	15.15 ± 0.06	8.85 ± 0.14	41.67 ± 5.39b	58.33 ± 5.39	73.16 ± 1.90	47.61 ± 9.00	65.16 ± 12.80
C1	14	26.17 ± 1.47	19.77 ± 1.24	15.11 ± 0.04	9.40 ± 0.31	42.00 ± 3.41	58.00 ± 3.41	71.67 ± 1.37	54.19 ± 3.17	75.67 ± 5.29
C2	14	25.83 ± 1.47	19.52 ± 1.96	15.10 ± 0.05	9.24 ± 0.13	45.50 ± 4.72	54.50 ± 4.72	71.41 ± 1.25	54.01 ± 5.39	75.68 ± 7.78
S1	14	26.33 ± 1.21	20.97 ± 0.80	15.12 ± 0.02	9.36 ± 0.28	43.83 ± 2.64	56.17 ± 2.64	71.14 ± 1.81	56.68 ± 2.03	79.69 ± 3.02
S2	14	26.83 ± 1.17	19.88 ± 0.55	15.13 ± 0.04	9.31 ± 0.11	42.17 ± 2.56	57.83 ± 2.56	71.87 ± 1.26	53.28 ± 1.08	74.15 ± 1.40
S3	14	26.17 ± 0.75	19.83 ± 1.43	15.12 ± 0.02	9.31 ± 0.10	45.67 ± 3.88	54.83 ± 4.02	71.04 ± 1.33	53.82 ± 3.33	75.73 ± 3.62
C1	21	30.33 ± 10.15	18.47 ± 0.79b	15.20 ± 0.25	9.57 ± 0.11	41.67 ± 1.51ab	56.17 ± 2.23	74.67 ± 5.63	48.66 ± 12.00b	66.43 ± 20.75b
C2	21	30.33 ± 6.83	19.40 ± 1.91ab	15.21 ± 0.15	9.52 ± 0.10	42.17 ± 3.87ab	56.00 ± 3.95	74.72 ± 3.44	49.23 ± 9.60b	66.39 ± 15.06b
S1	21	30.00 ± 7.04	19.95 ± 2.70ab	15.20 ± 0.18	9.53 ± 0.12	41.17 ± 1.47b	56.33 ± 1.63	74.32 ± 4.64	51.12 ± 10.81b	69.56 ± 17.86b
S2	21	26.33 ± 3.78	18.38 ± 1.51b	15.11 ± 0.11	9.56 ± 0.20	45.33 ± 4.80ab	55.33 ± 4.50	72.19 ± 3.37	51.12 ± 7.14b	71.29 ± 13.32b
S3	21	25.33 ± 2.16	21.70 ± 2.31a	15.09 ± 0.06	9.41 ± 0.12	47.67 ± 5.57a	51.00 ± 5.83	70.94 ± 2.22	62.95 ± 9.27a	88.70 ± 12.63a

	ANOVA (*p*-values)								
Age (A)	0.14	0.00	0.29	0.00	0.06	0.07	0.03	0.16	0.09
Supplementation (S)	0.51	0.00	0.54	0.81	0.19	0.27	0.07	0.30	0.36
AxS	0.07	0.01	0.22	0.17	0.02	0.14	0.10	0.04	0.02

*Erythrocytes transformed to natural logarithm; Different letters indicate significant difference FDR correction (*p* < 0.05); C1: base diet; C2: base diet + 50 ppm of zinc bacitracin; S1: 5.63 mg/Kg BW, S2: 11.0 mg/Kg BW, and S3: 16.3 mg/Kg BW of EEMC; HTO: hematocrit; HB: hemoglobin; ERY: erythrocytes; LEU: leukocytes; LYN: lymphocytes; GRA: granulocytes; MCV: mean corpuscular volume; MCH: mean corpuscular hemoglobin; MCHC: mean corpuscular hemoglobin concentration.

### Antimicrobial activity *in vivo*

**Figure 2:**
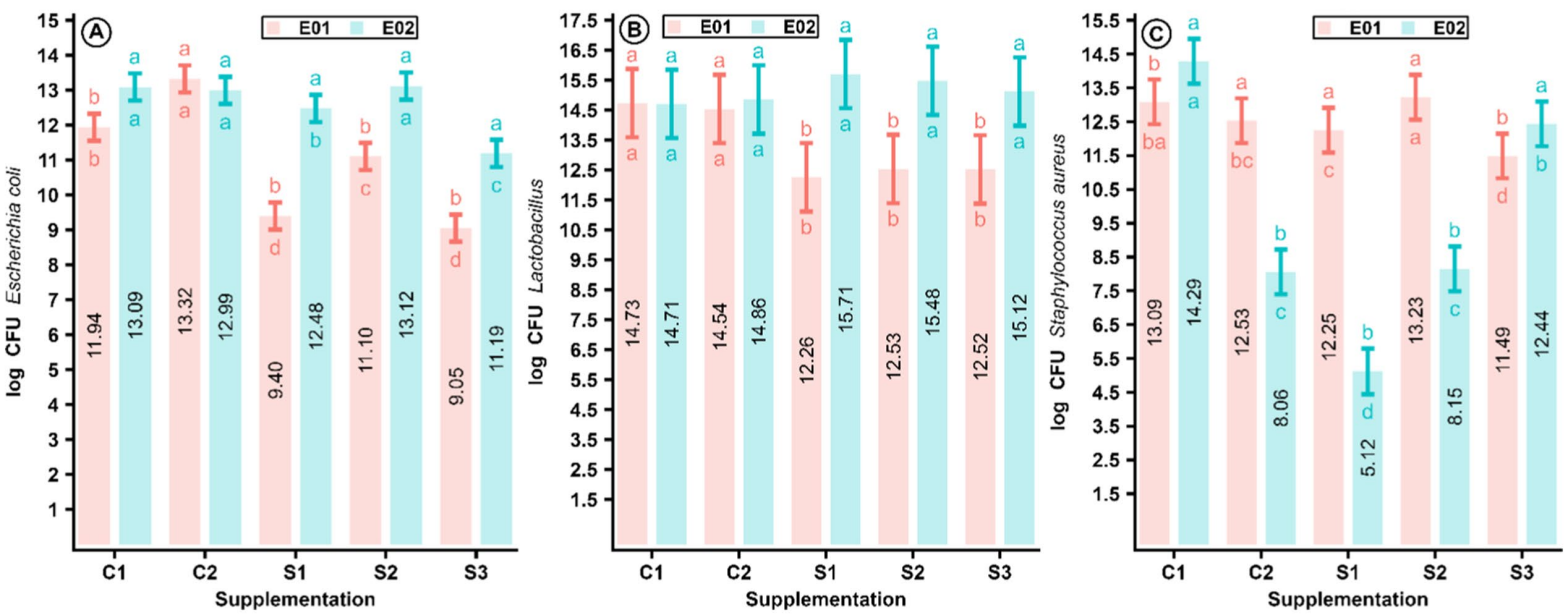
Interaction of MCEE supplementation and chickens age on *Escherichia coli* **(A)**, *Lactobacillus* sp. **(B)**, and *Staphylococcus aureus* abundance **(C)** (logCFU/g) in ileum content on supplementation with daily doses of 5.62, 11.0, and 16.3 mg/Kg BW of MCEE (*n* = 15), (CI = 95%). abc: Different letters at the bottom of intervals denotes dependance of MCEE supplementations on chicken age (*p* = 0.01), abc: different letter at the top of intervals denotes dependance of chicken age on MCEE supplementation (*p* = 0.01). CFU: Colony forming units. C1 (−): Base diet, C2 (+): Base diet + zinc bacitracin, S1: 5.62 mg/Kg BW, S2: 11.0 mg/Kg BW, S3: 16.3 mg/Kg BW of MCEE. E01: 21 days of age, E02: 28 days of age.

The microbial population obtained from the content and mucosa of the ileum of broiler chickens at 21 and 28 days of age, expressed as log (CFU/g) of fresh intestinal content, is shown in Supplementary material Table 4 and in Figure 2. This figure illustrates that the effect of MCEE supplementation on the abundance of *E. coli*, *S. aureus*, and *Lactobacillus* sp. in the chickens’ microbiota depends on the chickens’ age, while the abundance of these microorganisms is also influenced by the MCEE supplementation. The abundance log (CFU/g) of *E. coli* decreased in the S1, S2, and S3 groups of chickens supplemented with daily doses of 5.62, 11.0, and 16.3 mg/Kg BW EEMC at 21 days of age. Nonetheless, this reduction was maintained at 28 days of age for chickens supplemented with 11.0 and 16.3 mg/Kg BW EEMC, compared to group C1 (*p* < 0.001) (Figure 2A). Similarly, the abundance log (CFU/g) of *S. aureus* decreased in the S1 and S3 groups of chickens at 21 days of age, and this reduction persisted at 28 days of age. This abundance was also reduced in the S2 group of chickens compared to the C1 group (*p* < 0.001) (Figure 2C). In line with the abundance of *E. coli* and *S. aureus*, the abundance log (CFU/g) of *Lactobacillus* sp. was also reduced in the S1, S2, and S3 groups of chickens at 21 days of age (*p* < 0.001); however, this abundance recovered to a level similar to that of the C1 group at 28 days of age (*p* > 0.05) (Figure 2B).

### Gut histology

**Figure 3:**
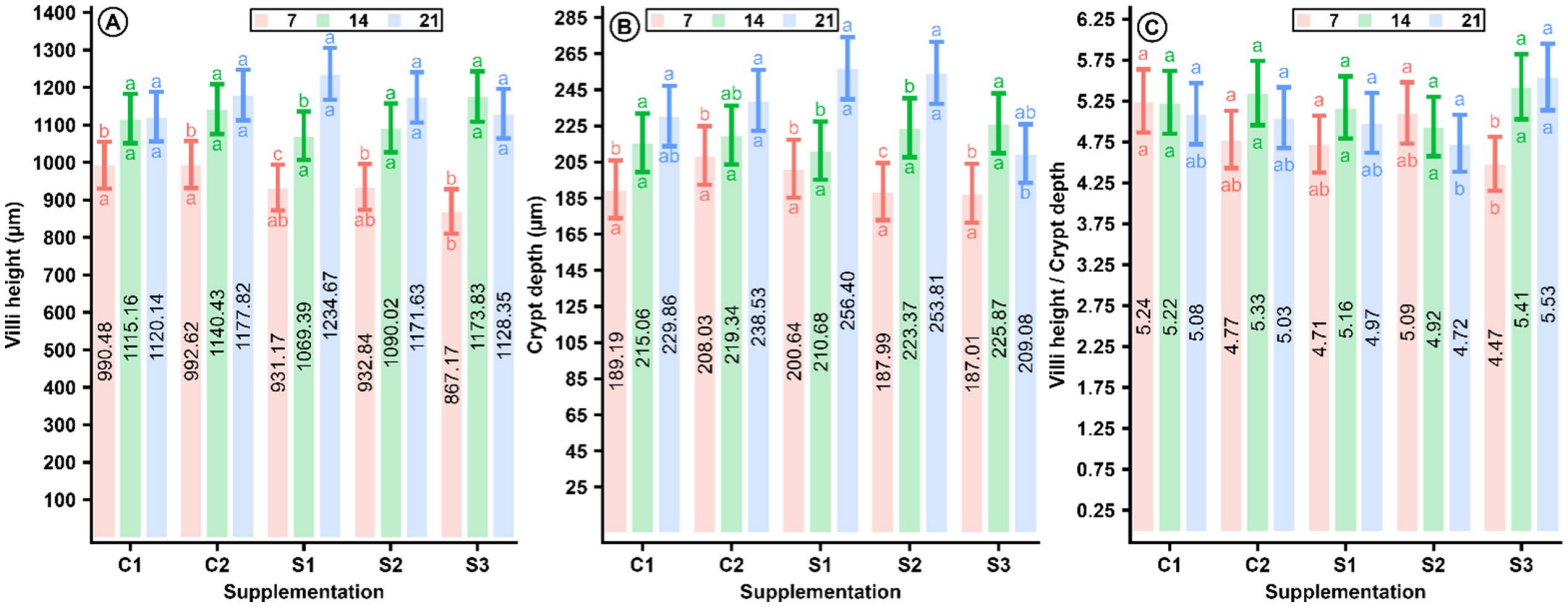
Interaction of MCEE supplementation and chickens age on villi hight **(A)**, crypt’s depth **(B)**, and villi height to crypt’s depth **(C)** as an average of the three segments on supplementation with daily doses of 5.62, 11.0, and 16.3 mg/kg BW of MCEE (*n* = 30), (CI = 95%). Abc: different letters at the bottom of intervals denotes dependance of MCEE supplementations on chicken age (*p* ≤ 0.02). abc: different letters at the top of intervals denotes dependance of chicken age on MCEE supplementation (*p* ≤ 0.02). C1 (−): base diet, C2 (+): base diet + zin bacitracin, S1: 5.62 mg/kg BW, S2: 11.0 mg/kg BW, S3: 16.3 mg/kg BW of MCEE.

Measurements of villus height, crypt depth, and villus width as average measurements in the duodenum, jejunum, and ileum of chickens are presented in Supplementary material Table 3 and in Figure 1, where these villus characteristics in the three segments indicate a healthy mucosal structure. The interaction of the MCEE effect and chicken age on villus height, crypt depth, and villus height-to-crypt depth ratio as an average of the three segments is depicted in Figure 3. This figure shows that intestinal villus height, crypt depth, and villus height-to-crypt depth ratio in chickens depend on MCEE supplementation. Villus height increased with MCEE supplementation in the S1 group of chickens at 21 days of age compared to the villus height obtained in C1, C2, S2, and S3 (*p* = 0.01) (Figure 3A).

Similarly, crypt depth increased in the S2 group at 21 days of age compared to the depth obtained in all other groups of chickens (*p* = 0.02) (Figure 3B). Additionally, villus height-to-crypt depth ratio increased with MCEE supplementation in the S3 group of chickens at 21 days of age (*p* = 0.01) (Figure 3C).

### Growth performance

The weight gain, feed conversion rate, and feed intake indices were evaluated for the initial, growth, and fattening stages, as well as the overall three phases for the broiler chickens as the primary indices for assessing animal production performance (25). In Supplementary material Tables 1, 2 and Table 5, the performance indices affected by supplementation with daily doses of 5.62, 11.0, and 16.3 mg/Kg BW MCEE in the diet, administered to broiler chickens in drinking water, are presented.

**Table 5:** Performance indices of broiler chickens supplemented ethanolic extract from *Morinda citrifolia* leaves (Mean ± SD) (*n* = 350).

Performance indices	Stage	C1	C2	C1/C2 (%)	Supplementation	S2	S3	ANOVA	CV (%)	Adjusted *R*^2^ (%)
					S1			*p*-value		
Feed intake (FI) (g)	Initial	144.83 ± 4.51	147.44 ± 4.49	98.22	145.65 ± 10.03	150.92 ± 2.11	148.08 ± 6.46	0.48	4.16	0.00
	Growth	814.06 ± 21.11	834.78 ± 38.38	97.53	805.25 ± 31.61	822.38 ± 17.96	782.13 ± 50.25	0.13	4.24	11.96
	Fattening	1924.96 ± 51.31	1957.31 ± 98.73	98.34	1910.28 ± 80.01	1939.35 ± 60.20	1949.47 ± 93.75	0.85	4.07	0.00
	Overall	2883.85 ± 42.93	2939.52 ± 101.88	98.11	2861.18 ± 102.60	2912.65 ± 68.42	2879.68 ± 138.31	0.65	3.30	0.00
Weight gain (g)	Initial	122.81 ± 5.79	123.79 ± 5.35	99.21	117.70 ± 10.41	125.26 ± 6.76	124.35 ± 6.98	0.42	5.93	0.15
	Growth	533.39 ± 10.74	542.51 ± 41.11	98.34	529.55 ± 35.88	548.53 ± 15.88	524.12 ± 28.52	0.60	5.38	0.00
	Fattening	1142.99 ± 38.17b	1197.37 ± 35.34ab	95.46	1147.35 ± 48.97b	1233.58 ± 31.18a	1173.07 ± 57.79ab	0.03	4.45	22.27
	Overall	1799.19 ± 42.59b	1863.67 ± 29.85ab	96.54	1794.60 ± 42.25b	1907.37 ± 33.39a	1821.53 ± 80.58ab	0.01	3.05	31.72
Feed conversion rate (FCR)	Initial	1.18 ± 0.05	1.19 ± 0.07	99.16	1.24 ± 0.06	1.21 ± 0.05	1.19 ± 0.04	0.40	4.55	0.72
	Growth	1.53 ± 0.06	1.54 ± 0.07	99.35	1.52 ± 0.08	1.50 ± 0.04	1.49 ± 0.07	0.67	4.25	0.00
	Fattening	1.69 ± 0.06	1.64 ± 0.08	100.03	1.67 ± 0.06	1.57 ± 0.07	1.66 ± 0.09	0.16	4.92	10.10
	*Overall	1.61 ± 0.04a	1.58 ± 0.05a	100.02	1.59 ± 0.05a	1.53 ± 0.04b	1.58 ± 0.05ab	0.03		

abc, Different letters indicate significant difference, FDR correction (*p* < 0.05); C1: base diet; C2: base diet + 50 ppm of zinc bacitracin; S1: 5.63 mg/Kg BW, S2: 11.0 mg/Kg BW, and S3: 16.3 mg/Kg BW of EEMC, VC: variation coefficient.

Final weight and weight gain during the fattening phase, as well as overall weight gain, were greater in chickens from the group supplemented with 11.0 mg/Kg BW MCEE (S2) (*p* ≤ 0.03). Following a similar trend as the two previous indices, the feed conversion rate was overall lower in the chickens supplemented with 11.0 mg/Kg BW of MCEE (S2) than in the C1 and C2 groups (*p* ≤ 0.03). Additionally, it was lower than the feed conversion rates observed for the chickens supplemented with 5.6 mg/Kg BW (S1) and 16.3 mg/Kg BW (S3) of MCEE (*p* = 0.03) (Table 5). Chicken mortality was recorded at 1.10% (4 chickens) of the lot.

## Discussion

Polyphenols, flavonoids, tannins, triterpenes, and steroids were the main phytocompounds of noni leaf obtained in the present study (12, 13), and these results differ in very few phytocompounds from those obtained in noni fruit in previous studies, in which triterpenes and steroids were not detected (69). However, alkaloids have been identified as secondary metabolites in noni fruit (29). The variation in the phytochemical content of noni is attributed to the diversity of geographical and environmental factors, such as soil, solar light, temperature, and precipitation, as well as post-growth factors, such as harvest, storage, transportation, and processing (29). This might explain the improvement in growth performance in broiler chickens concerning the effect of MCEE in the present study compared with those obtained in two previous studies where no variation in these indices was reported (14, 15).

### Hematological profiles

Studies investigating the effects of *M. citrifolia* extracts or essential oils on the hematological and blood metabolite profiles of birds are limited. Blood is the primary tissue that produces cells responsible for transporting oxygen, which powers cellular metabolism (30, 31). The increase in hemoglobin, MCH, and MCHC in chickens at 21 days of age due to the effect of MCEE in the present study has also been reported in a previous study, where hemoglobin, MCH, and MCHC showed a quadratic trend as the level of MCEE increased from 0, 50, to 100 ppm in the diet for broiler chickens at 28 days of age (2). Similar results have been obtained in previous studies where guinea pigs were supplemented with 4% noni fruit powder, leading to increased levels of MCH and MCHC (10). Hemoglobin facilitates the delivery of oxygen to tissues where it is needed, as oxygen is consumed; therefore, hemoglobin levels are associated with metabolic health and body mass index (30–32). The increase in hemoglobin, MCH, and MCHC in chickens at 21 days of age in the present study may be attributed to the chickens’ response to phytochemical compounds such as phenolic compounds, flavonoids, amino acids, steroids, and triterpenes identified in the phytochemical screening, which may confer the antioxidant activity of the MCEE (6, 33–38, 70). This is supported by the results of the present study, which showed that MCEE inhibited 58% of the DPPH radicals with a CI_50_ of 137.89 μg/mL.

### Antimicrobial activity *in vivo*

The microbial population was obtained from the mucosa of the ileum because protein and starch digestion occurs, with approximately 95% reaching the terminal ileum (71). Furthermore, ileal microbiota contribute to starch digestion (8), and in the chicken ileal microbiota, Firmicutes (64.15%), Bacteroidetes (22.15%), and Proteobacteria (4.26%) predominate (39). In addition, the total amount of ileal bacteria can be modified by probiotics (40), antibiotic growth promoters (9), and plant essential oils and extracts (3, 22), which appear to be more effective in the hind segments of the intestinal tract (41).

Most of the microbes in the intestinal microbiota of poultry identified in cultivation-based studies are Gram-positive rods and cocci (86%), followed by Gram-negative rods (14%) (42, 43). However, more recent studies using 16S rRNA methodology reveal that the chicken intestinal microbiota predominantly consists of the phyla: Firmicutes (50%), Cyanobacteria (26%), and Proteobacteria (17%) (43, 44). Furthermore, the predominance of one phylum of bacteria, among other factors, is associated with the gender and breed of chickens (45). This gut microbiota can be regulated by different groups of additives, including essential oils and extracts (46, 47). The antimicrobial activity of an extract or essential oil from a plant depends on various factors, including the chemical structure of its components (48). In this regard, it is primarily attributed to the polyphenol content, and their concentration determines the antimicrobial potential of plants (49–51).

A decrease in the abundance log(CFU/g) of *S. aureus* in the intestinal content of broiler chickens due to the effect of MCEE at 5.62, 11.0, and 16.3 mg/Kg BW from 1 to 21 days of age in the present study concurred with the results obtained in the minimum inhibitory concentration (MIC) test in our previous study, in which MCEE inhibited the growth of the ATCC 25923 strain of *S. aureus* at a concentration of 3.12 mg/mL. Simultaneously, this result was consistent with that obtained in a previous study, where 100 ppm MCEE supplemented in drinking water reduced the abundance of *S. aureus* by 21 days in broiler chickens (2). However, a decrease in the abundance log(CFU/g) of *E. coli* in the groups of chickens supplemented with daily doses of 5.62, 11.0, and 16.3 mg/Kg BW MCEE at 21 days of age in the present study differs from a previous study where MCEE at 100 ppm did not reduce the abundance of *E. coli* and *Lactobacillus* sp. in the gut of chickens (2), marking this as the first report of the antimicrobial activity *in vivo* of MCEE on *E. coli* in chickens. The maintenance of the reduction in *S. aureus* and *E. coli* and the enhancement of the abundance of *Lactobacillus* sp. at 28 days of age, despite the lack of MCEE supplementation from 21 to 28 days, might be related to a selective residual effect of MCEE phytocompounds on *S. aureus* and *E. coli* but not on *Lactobacillus* sp. in the gut microbiota of broiler chickens. This could be explained by the fact that most lactobacilli exhibit intrinsic resistance to several antibiotics (52, 53), especially to residual concentrations in the gut, as is the case with the levels of MCEE’s phytocompounds present in the chickens at 28 days old in this study.

This highlights the effect of MCEE in modulating the gut health of chickens since gram-positive bacteria are the most abundant in the gut (42, 43), and gram-negative bacteria release greater levels of endotoxins than gram-positive bacteria, which induce inflammation of the epithelial gut mucosa, affecting intestinal health to a greater extent (54). *Lactobacillus* sp. is a beneficial bacterium in the gut.

Similar results have also been obtained in previous studies on the in vivo antimicrobial activity of extracts, essential oils, and phytogenic additives from other plants, where a reduction in *E. coli* abundance in the intestinal content of chickens has been reported (1, 21, 22, 55, 56).

Therefore, the reduction in the abundance log (UFC/g) of *E. coli* and *S. aureus* and the maintenance of *Lactobacillus* sp. in the intestinal microbiota of broiler chickens due to MCEE in the present study highlights the wide spectrum and selective antimicrobial activity of the components from these groups of phytocompounds in MCEE. Similar results have been found in previous studies, where noni fruit phenolic extract ameliorated gut microbiota dysbiosis in mice by increasing short-chain fatty acid-producing bacteria and decreasing lipopolysaccharide-producing bacteria, revealing the relative abundance of the predominant taxa at phylum and genus levels, in particular the special taxa responding to noni fruit phenolic extract (8).

In addition, noni fruit phenolic extract also restored the composition of gut microbiota in mice, with a remarkable elevation in the relative abundance of Parabacteroides, Lactobacillus, Roseburia, and Akkermansia, alongside a significant reduction in Helicobacter, norank_f_Desulfovibrionaceae, Desulfovibrio, and Mucispirillum at the genus level (8).

In another previous study, noni fruit polysaccharide administration in mice reversed the induced increases in the relative abundances of Coprococcus, Odoribacter, and Alistipes. In addition, these polysaccharides also reversed the induced decreases in the relative abundances of Lactobacillus, Anaerotruncus, and Bilophila (57).

These findings support the potential of MCEE phytocompounds in modulating gut microbiota in different animal species, which will be highly beneficial for gut health and improving animal health and production.

### Gut histology

The mechanisms for increasing the number of cells and activity in the mucosa of the duodenum, jejunum, and ileum are cornerstones of gut health, which can improve as the crypts increase in depth (58, 59).

In the present study, this increase was age-dependent upon supplementation with 11 mg/Kg BW of MCEE in chickens at 21 days of age, where the intestinal crypts had the greatest depth compared with the results obtained for chickens from the C1 and C2 groups (*p* = 0.02). This result aligns with a previous study in which supplementing broiler chickens with a daily 100 ppm of MCEE increased crypt depth with age (2).

The increase in the villus height to crypt depth ratio is also dependent on age upon supplementation with a daily dose of 16.3 mg/Kg BW MCEE, which is consistent with previous studies on essential oils or extracts from other plants (38, 58–60). An increase in these mucosal structures promotes the presence of Paneth cells in birds (61), which secrete antimicrobial products such as lysozymes, and enteroendocrine cells, which secrete local hormones and increase the population of mucus-secreting goblet cells (62, 63) in a balanced manner.

This increase also improves nutrient absorption and enzyme production, owing to a more dynamic replacement mechanism for enterocytes in the villi.

Furthermore, because of the MCEE, the development of these mechanisms in the intestinal mucosa observed in the present study might be associated with antimicrobial (64–66) and antioxidant activities (33, 34, 37).

The high level of antioxidant activity of *M. citrifolia* fruit juice, which is 2.8 times that of vitamin C, diminishes blood levels of malondialdehyde and enhances the antioxidant defense system of superoxide dismutase (33, 34, 37, 38). These mechanisms could promote the proliferation and growth of crypts that originate from pluripotent cells of different cellular groups in the intestinal mucosa and increase the villus height and villus height-to-crypt depth ratio observed in the present study. To the best of our knowledge, this is the first study to demonstrate the effect of MCEE on gut histology modulation.

### Growth performance

Few studies have investigated the effects of *M. citrifolia* extracts or essential oils on the growth performance of broiler chickens (2, 14, 15). An increase in weight gain and a reduction in feed conversion rate are the primary indices used when evaluating performance in animal production (25).

The improvement in total weight, weight gain, and feed conversion rate during the fattening phase, and overall, in broiler chickens supplemented with a daily dose of 11.0 mg/Kg BWMCEE in the diet in the present study may be associated with antimicrobial compounds that have potent inhibitory activity on the intestinal microbiota, thereby modulating it, as shown in this study, which might strengthen the intestinal health of broiler chickens (67).

This could also be related to an increase in hemoglobin, MCH, and MCHC profiles due to their antioxidant properties (6, 35), thus improving tissue oxygenation and metabolic activity (30, 31). At the same time, the liver is protected from injury by some bioactive compounds in this plant (38, 68), and this mechanism might enhance the accumulation of protein in broiler chicken tissue, similar to that observed in Nile tilapia (11).

The results of the present study are similar to those obtained in previous studies, which reported an improvement in the growth performance of tilapia, guinea pigs, and broiler chickens using fruit extract, fruit powder, and leaf extract of noni, respectively (2, 10, 11). However, the results in the present study contrast with those obtained in other studies, which found no changes in the growth performance of chickens with the use of noni fruit flour at different levels or fruit extract from this plant (14, 15); additionally, the MCEE did not show any effect on daily feed consumption (*p* < 0.05).

These contrasting results may be due to the variation in the phytochemical content of *M. citrifolia* caused by factors such as soil, sunlight, temperature, and precipitation, which vary according to geographic location (29).

We also acknowledge the challenges that arise in studying changes in chicken microbiota using 16S rRNA methodology, which provides in-depth determination of microbiota communities at the phyla and genus levels in animal species. This methodology has shown that the intestinal microbiota of chickens predominantly consists of the phyla: Firmicutes (50%), Cyanobacteria (26%), and Proteobacteria (17%) (43, 44). These microbiota communities change dynamically as diet composition varies; therefore, in this study, MCEE may have influenced these changes in chicken microbiota, which remain to be explored.

## Conclusion

Supplementing broiler chickens with a daily dose of 11.0 mg/Kg BW MCEE decreased the abundance of *E. coli*, *S. aureus*, and *Lactobacillus* sp. in the intestinal microbiota, while villus height and crypt depth increased with age at 21 days, indicating that MCEE improved intestinal health. In addition, daily doses of 16.3 mg/Kg BW of MCEE increased hemoglobin, MCH, and MCHC levels at 21 days. The interactions between improved intestinal health and increased hemoglobin profiles may have enhanced the final weight, weight gain, and feed conversion rate during the fattening stage. Thus, this study demonstrated the beneficial effect of MCEE as a supplement for improving gut health and performance indices in broiler chickens. Furthermore, MCEE did not adversely affect physiological indices, suggesting it could be a promising candidate, pending further validation, as an alternative to AGP in poultry. However, further studies are needed to elucidate the phytocompounds in MCEE and the mechanisms underlying these beneficial effects.

## Data Availability

The raw data supporting the conclusions of this article will be made available by the authors, without undue reservation.
