# Supplementary material for: Ethanolic extract of Morinda citrifolia improves gut microbiota, intestinal morphology, and performance without adverse effects on hematological profiles in broiler chickens
Source: Front Vet Sci. 2026 Jan 28;12:1686136. doi: 10.3389/fvets.2025.1686136 (PMC12892492; doi:10.3389/fvets.2025.1686136)
Supplement: Supplementary file 1 [file Data_Sheet_1.pdf]

# SM TABLE 1: DATA OF FEED INTAKE FROM CHICKENS SUPPLEMENTED 5.63, 11.0 and 16.3 MG/KG BODY WEIGHT DAILY OF MORINDA CITRIFOLIA ETHANOLIC EXTRACT

C

| Feed (g) | | | | | | | | | | | | | | | | |
|---|---|---|---|---|---|---|---|---|---|---|---|---|---|---|---|---|
| | feed for Initial phase (g) (1-7 days) | | | | | | feed for growth phase (g) (8-21 days) | | | | | feed for fattening phase (g) (22-35 days) | | | | |
| Supplementation/replicate | Offered feed (g) | Remaining feed in bag (g) | Remaining feed in feeder (g) | Total remaining feed (g) | Feed intake/pen (g) | Feed intake/chicken (g) | Offered feed (g) | Remaining feed in bag (g) | Remaining feed in feeders (g) | Total remaining feed (g) | Feed intake /pen (g) | Offered Feed (g) | Remaining feed in bag (g) | Remaining feed in feeder (g) | Total feed remaining (g) | Feed intake/pen (g) |
| C1R1 | 2500 | 804 | 111 | 915 | 1585 | 144.09 | 12500 | 3938 | 288 | 4226 | 8274 | 18200 | 1636 | 504 | 2140 | 16060 |
| C1R2 | 2500 | 784 | 151 | 935 | 1565 | 142.27 | 12500 | 3784 | 37 | 3821 | 8679 | 19000 | 2485 | 413 | 2898 | 16102 |
| C1R3 | 2500 | 798 | 85 | 883 | 1617 | 147.00 | 12500 | 3334 | 645 | 3979 | 8521 | 18200 | 1516 | 586 | 2102 | 16098 |
| C1R4 | 2500 | 837 | 114 | 951 | 1549 | 140.82 | 12500 | 3284 | 591 | 3875 | 8625 | 19000 | 1858 | 421 | 2279 | 16721 |
| C1R5 | 2500 | 843 | 96 | 939 | 1561 | 141.91 | 12500 | 3994 | 320 | 4314 | 8186 | 19000 | 1588 | 370 | 1958 | 17042 |
| C1R6 | 2500 | 689 | 129 | 818 | 1682 | 152.91 | 12500 | 3202 | 579 | 3781 | 8719 | 19000 | 2563 | 443 | 3006 | 15994 |
| C2R1 | 2500 | 677 | 198 | 875 | 1625 | 147.73 | 12500 | 3330 | 465 | 3795 | 8705 | 17300 | 0 | 479 | 479 | 16821 |
| C2R2 | 2500 | 658 | 311 | 969 | 1531 | 139.18 | 12500 | 2942 | 66 | 3008 | 9492 | 16500 | 0 | 592 | 592 | 15908 |
| C2R3 | 2500 | 710 | 110 | 820 | 1680 | 152.73 | 12500 | 3198 | 386 | 3584 | 8916 | 17300 | 0 | 301 | 301 | 16999 |
| C2R4 | 2500 | 692 | 175 | 867 | 1633 | 148.45 | 12500 | 3080 | 779 | 3859 | 8641 | 16500 | 0 | 558 | 558 | 15942 |
| C2R5 | 2500 | 707 | 173 | 880 | 1620 | 147.27 | 12500 | 3614 | 496 | 4110 | 8390 | 16500 | 0 | 503 | 503 | 15997 |
| C2R6 | 2500 | 660 | 198 | 858 | 1642 | 149.27 | 12500 | 3868 | 185 | 4053 | 8447 | 16500 | 0 | 470 | 470 | 16030 |
| S1R1 | 2500 | 561 | 437 | 998 | 1502 | 136.55 | 12500 | 4018 | 559 | 4577 | 7923 | 20000 | 2882 | 666 | 3548 | 16452 |
| S1R2 | 2500 | 765 | 271 | 1036 | 1464 | 133.09 | 12500 | 4028 | 81 | 4109 | 8391 | 20000 | 3662 | 613 | 4275 | 15725 |
| S1R3 | 2500 | 629 | 230 | 859 | 1641 | 149.18 | 12500 | 4074 | 175 | 4249 | 8251 | 20000 | 3870 | 768 | 4638 | 15362 |
| S1R4 | 2500 | 808 | 99 | 907 | 1593 | 144.82 | 12500 | 3722 | 130 | 3852 | 8648 | 20000 | 2952 | 481 | 3433 | 16567 |
| S1R5 | 2500 | 664 | 194 | 858 | 1642 | 149.27 | 12500 | 3454 | 284 | 3738 | 8762 | 20000 | 3462 | 501 | 3963 | 16037 |
| S1R6 | 2500 | 674 | 55 | 729 | 1771 | 161.00 | 12500 | 3044 | 700 | 3744 | 8756 | 20000 | 2304 | 415 | 2719 | 17281 |
| S2R1 | 2500 | 602 | 230 | 832 | 1668 | 151.64 | 12500 | 3448 | 430 | 3878 | 8622 | 20000 | 2419 | 603 | 3022 | 16978 |
| S2R2 | 2500 | 769 | 81 | 850 | 1650 | 150.00 | 12500 | 3140 | 765 | 3905 | 8595 | 20000 | 3436 | 509 | 3945 | 16055 |
| S2R3 | 2500 | 679 | 181 | 860 | 1640 | 149.09 | 12500 | 3710 | 279 | 3989 | 8511 | 20000 | 2796 | 923 | 3719 | 16281 |
| s2R4 | 2500 | 677 | 121 | 798 | 1702 | 154.73 | 12500 | 3782 | 149 | 3931 | 8569 | 20000 | 3318 | 772 | 4090 | 15910 |
| s2R5 | 2500 | 730 | 100 | 830 | 1670 | 151.82 | 12500 | 3120 | 362 | 3482 | 9018 | 20000 | 3018 | 1168 | 4186 | 15814 |
| s2R6 | 2500 | 727 | 130 | 857 | 1643 | 149.36 | 12500 | 3144 | 565 | 3709 | 8791 | 20000 | 3989 | 836 | 4825 | 15175 |
| S3R1 | 2500 | 914 | 12 | 926 | 1574 | 143.09 | 12500 | 4050 | 127 | 4177 | 8323 | 20000 | 2097 | 650 | 2747 | 17253 |
| S3R2 | 2500 | 846 | 28 | 874 | 1626 | 147.82 | 12500 | 3100 | 380 | 3480 | 9020 | 20000 | 2734 | 396 | 3130 | 16870 |
| S3R3 | 2500 | 405 | 378 | 783 | 1717 | 156.09 | 12500 | 3996 | 60 | 4056 | 8444 | 20000 | 2547 | 395 | 2942 | 17058 |
| S3R4 | 2500 | 842 | 130 | 972 | 1528 | 138.91 | 12500 | 4516 | 265 | 4781 | 7719 | 20000 | 4071 | 602 | 4673 | 15327 |
| S3R5 | 2500 | 720 | 86 | 806 | 1694 | 154.00 | 12500 | 2806 | 0 | 2806 | 9694 | 20000 | 2534 | 372 | 2906 | 17094 |
| S3R6 | 2500 | 685 | 181 | 866 | 1634 | 148.55 | 12500 | 4380 | 564 | 4944 | 7556 | 20000 | 3659 | 520 | 4179 | 15821 |

Means

| | | | | | | | | | | | | | | | | |
|---|---|---|---|---|---|---|---|---|---|---|---|---|---|---|---|---|
| C1 | 2500 | 792.50 | 114.33 | 906.83 | 1593.17 | 144.83 | 12500 | 3589.33 | 410 | 3999.33 | 8500.67 | 18733.33 | 1941 | 456.17 | 2397.17 | 16336.17 |
| C2 | 2500 | 684.00 | 194.17 | 878.17 | 1621.83 | 147.44 | 12500 | 3338.67 | 396.17 | 3734.83 | 8765.17 | 16766.67 | 0 | 483.83 | 483.83 | 16282.83 |
| S1 | 2500 | 683.50 | 214.33 | 897.83 | 1602.17 | 145.65 | 12500.00 | 3723.33 | 321.50 | 4044.83 | 8455.17 | 20000.00 | 3188.67 | 574.00 | 3762.67 | 16237.33 |
| S2 | 2500 | 697.33 | 140.50 | 837.83 | 1662.17 | 151.11 | 12500.00 | 3390.67 | 425.00 | 3815.67 | 8684.33 | 20000.00 | 3162.67 | 801.83 | 3964.50 | 16035.50 |
| s3 | 2500 | 735.33 | 135.83 | 871.17 | 1628.83 | 148.08 | 12500.00 | 3808.00 | 232.67 | 4040.67 | 8459.33 | 20000.00 | 2940.33 | 489.17 | 3429.50 | 16570.50 |

C1: base diet, C2: Base diet +50 ppm Zin bacitracin, S1: supplemented 5.63 mg/kg BW, S2: supplemented 11.0 mg/Kg BW, S3: supplemented 16.3 mg/Kg BW MCEE
