# Supplementary material for: Ethanolic extract of Morinda citrifolia improves gut microbiota, intestinal morphology, and performance without adverse effects on hematological profiles in broiler chickens
Source: Front Vet Sci. 2026 Jan 28;12:1686136. doi: 10.3389/fvets.2025.1686136 (PMC12892492; doi:10.3389/fvets.2025.1686136)
Supplement: Supplementary file 2 [file Data_Sheet_2.pdf]

SM TABLE 2: DATA OF WEIGHT FROM CHICKENS SUPPLEMENTED 5.63, 11.0 and 16.3 MG/KG BODY WEIGHT DAILY OF MORINDA CITRIFOLIA ETHANOLIC EXTRACT

Weight of control 1 (C1)							
			Day 1	Day 7	Day 14	Day 21	Day 35
Trat.	Rep.	Num.	Weight (g)	Weight (g)	Weight (g)	Weight (g)	Weight (g)
C1	R1	1	38	153	452	788	1992
C1	R1	2	35	158	420	764	1828
C1	R1	3	37	166	385	718	1373
C1	R1	4	36	169	425	680	2043
C1	R1	5	36	151	353	658	1848
C1	R1	6	43	157	380	738	1868
C1	R1	7	41	150	411	668	1849
C1	R1	8	47	153	401	632	1902
C1	R1	9	45	148	342	534	
C1	R1	10	38	143	436	584	
C1	R1	11	33	156	349		
C1	R2	1	35	119	372	763	1917
C1	R2	2	33	159	437	674	1818
C1	R2	3	37	174	355	662	1878
C1	R2	4	34	169	409	728	1889
C1	R2	5	41	161	385	788	1798
C1	R2	6	36	170	357	670	1725
C1	R2	7	41	137	474	622	2005
C1	R2	8	41	186	412	760	1437
C1	R2	9	33	151	403	662	
C1	R2	10	38	139	373	604	
C1	R2	11	32	156	458		
C1	R3	1	41	144	406	774	2228
C1	R3	2	39	145	395	734	1743
C1	R3	3	37	144	393	800	1853
C1	R3	4	32	187	388	762	1843
C1	R3	5	39	172	465	626	2058
C1	R3	6	32	185	466	594	2043
C1	R3	7	38	150	475	644	1849
C1	R3	8	39	189	450	752	1800
C1	R3	9	38	177	411	658	
C1	R3	10	34	166	376	678	
C1	R3	11	36	152	380		
C1	R4	1	35	158	387	811	2103
C1	R4	2	46	150	423	666	1750
C1	R4	3	35	164	356	676	1953
C1	R4	4	37	164	414	560	1900
C1	R4	5	34	162	391	688	1926
C1	R4	6	38	156	389	714	1739
C1	R4	7	39	185	406	620	1868
C1	R4	8	33	179	447	678	1863
C1	R4	9	38	159	360	692	
C1	R4	10	34	169	433	746	
C1	R4	11	40	154	318		
C1	R5	1	33	158	428	804	2028
C1	R5	2	39	162	344	690	1768
C1	R5	3	36	164	411	676	1998
C1	R5	4	39	130	464	768	1910
C1	R5	5	37	150	377	642	1808
C1	R5	6	33	138	396	734	1938
C1	R5	7	35	174	458	660	1866
C1	R5	8	35	151	415	718	1731
C1	R5	9	36	135	408	714	
C1	R5	10	35	171	442	602	
C1	R5	11	37	130	446		
C1	R6	1	40	150	407	722	2170
C1	R6	2	33	162	452	788	1428
C1	R6	3	33	180	400	580	2238
C1	R6	4	36	169	409	768	1839
C1	R6	5	38	157	375	688	2246
C1	R6	6	35	154	396	770	1364
C1	R6	7	36	164	405	558	1892
C1	R6	8	40	158	454	676	1952
C1	R6	9	37	185	460	742	
C1	R6	10	37	163	380	680	
C1	R6	11	37	160	357		
Mean			36.98	159.11	406.09	692.50	

Weight of control 2 (C2)							
			Day 1	Day 7	Day14	Day 21	Day 35
Supp.	Rep.	Num.	Weight 1 (g)	Weight 2 (g)	Weight 3 (g)	Weight 4 (g)	Weight 5 (g)
C2	R1	1	35	155	350	700	1928
C2	R1	2	38	169	400	682	1894
C2	R1	3	35	171	416	708	2026
C2	R1	4	33	152	381	740	1935
C2	R1	5	34	155	424	628	1900
C2	R1	6	33	166	475	680	1871
C2	R1	7	38	135	443	514	1833
C2	R1	8	39	144	452	608	1448
C2	R1	9	39	169	374	758	
C2	R1	10	37	187	446	768	
C2	R1	11	38	166	358		
C2	R2	1	35	163	512	850	1990
C2	R2	2	38	168	533	894	1842
C2	R2	3	34	171	451	668	1932
C2	R2	4	36	138	466	716	2038
C2	R2	5	38	150	466	724	2108
C2	R2	6	36	141	372	798	1769
C2	R2	7	35	164	378	638	2092
C2	R2	8	38	172	406	772	1671
C2	R2	9	38	178	420	948	
C2	R2	10	33	195	372	816	
C2	R2	11	40	163	405		
C2	R3	1	34	167	421	787	2019
C2	R3	2	33	163	425	640	2097
C2	R3	3	37	195	396	656	1968
C2	R3	4	34	174	487	714	1880
C2	R3	5	35	158	466	688	1912
C2	R3	6	39	156	432	678	1819
C2	R3	7	34	143	415	760	1788
C2	R3	8	40	161	420	640	1848
C2	R3	9	43	152	365	708	
C2	R3	10	41	164	372	660	
C2	R3	11	37	159	369		
C2	R4	1	35	150	391	835	2280
C2	R4	2	34	155	337	706	1773
C2	R4	3	35	159	484	774	1960
C2	R4	4	35	179	421	748	1730
C2	R4	5	38	187	347	658	1937
C2	R4	6	39	146	416	832	1688
C2	R4	7	35	179	382	746	2094
C2	R4	8	33	160	494	658	
C2	R4	9	36	164	466	600	
C2	R4	10	35	161	370	586	
C2	R4	11	36	177	378		
C2	R5	1	34	148	392	783	2176
C2	R5	2	39	138	395	720	1923
C2	R5	3	37	151	332	550	1993
C2	R5	4	36	148	467	700	1985
C2	R5	5	35	156	413	532	2063
C2	R5	6	34	154	421	662	1782
C2	R5	7	35	162	358	640	1638
C2	R5	8	40	155	415	662	1648
C2	R5	9	36	154	335	728	
C2	R5	10	36	183	375	578	
C2	R5	11	34	169	412		
C2	R6	1	38	143	436	753	2208
C2	R6	2	35	148	424	810	2008
C2	R6	3	41	155	334	740	1779
C2	R6	4	37	134	468	636	1778
C2	R6	5	35	160	415	602	1957
C2	R6	6	36	185	342	704	1822
C2	R6	7	36	162	396	642	1609
C2	R6	8	38	154	363	536	1838
C2	R6	9	35	155	447	820	
C2	R6	10	39	114	358	676	
C2	R6	11	34	159	315		
Mean			36.33	160.12	408.59	702.63	

Weight of supplementation 1 (S1)							
			Day 1	Day 7	Day 14	Day21	Day 35
Supp	Rep.	Num.	Weight 1 (g)	Weight 2 (g)	Weight 3 (g)	Weight 4 (g)	Weight 5 (g)
S1	R1	1	33	142	379	681	1955
S1	R1	2	38	166	346	624	1772
S1	R1	3	34	179	368	572	1807
S1	R1	4	37	156	430	626	1864
S1	R1	5	39	159	344	618	1628
S1	R1	6	36	138	405	586	1940
S1	R1	7	38	136	390	592	1947
S1	R1	8	36	168	367	672	1824
S1	R1	9	34	128	415	582	
S1	R1	10	37	148	341	658	
S1	R1	11	36	128	350		
S1	R2	1	36	170	421	909	2118
S1	R2	2	37	147	407	678	1537
S1	R2	3	37	137	409	510	1685
S1	R2	4	35	159	452	802	1936
S1	R2	5	35	148	450	604	1826
S1	R2	6	40	130	351	640	1981
S1	R2	7	33	132	391	642	1676
S1	R2	8	41	127	364	694	1834
S1	R2	9	39	148	286	640	
S1	R2	10	34	88	360	724	
S1	R2	11	36	139	523		
S1	R3	1	37	143	406	801	2160
S1	R3	2	35	154	351	744	1709
S1	R3	3	33	149	380	832	1848
S1	R3	4	34	142	485	624	1774
S1	R3	5	38	162	359	638	1727
S1	R3	6	37	151	442	656	1720
S1	R3	7	37	158	377	602	1329
S1	R3	8	35	138	428	752	1781
S1	R3	9	34	152	421	626	
S1	R3	10	39	156	352	523	
S1	R3	11	35	141	377		
S1	R4	1	39	154	370	800	1958
S1	R4	2	39	151	445	790	2135
S1	R4	3	36	152	351	630	1799
S1	R4	4	41	178	408	640	1900
S1	R4	5	35	173	348	722	1881
S1	R4	6	37	144	481	830	1674
S1	R4	7	38	157	379	652	1740
S1	R4	8	35	178	428	624	1956
S1	R4	9	39	146	372	668	
S1	R4	10	38	171	360	670	
S1	R4	11	34	155	398		
S1	R5	1	38	163	443	766	1923
S1	R5	2	37	173	450	674	1723
S1	R5	3	38	158	363	850	2110
S1	R5	4	35	161	368	756	1860
S1	R5	5	34	171	486	810	1774
S1	R5	6	34	157	434	642	1782
S1	R5	7	36	137	428	676	1760
S1	R5	8	39	155	384	732	1943
S1	R5	9	38	142	382	754	
S1	R5	10	33	140	444	686	
S1	R5	11	36	177	402		
S1	R6	1	35	162	414	704	2000
S1	R6	2	35	181	390	728	1676
S1	R6	3	34	170	360	790	1794
S1	R6	4	38	166	417	614	1964
S1	R6	5	37	165	425	626	1779
S1	R6	6	34	167	418	726	1580
S1	R6	7	39	174	440	648	1984
S1	R6	8	37	176	413	692	1811
S1	R6	9	35	166	415	668	
S1	R6	10	35	181	439	594	
S1	R6	11	34	145	482		
Mean			36.32	154.02	400.97	683.57	

Weight of supplementation 2 (S2)							
			Day 1	Day 7	Day 14	Day 21	Day 35
Supp.	Rep.	Num.	Weight 1 (g)	Weight 2 (g)	Weight 3 (g)	Weight 4 (g)	Weight 5 (g)
S2	R1	1	33	170	454	760	2003
S2	R1	2	36	185	411	698	1762
S2	R1	3	33	143	386	848	1953
S2	R1	4	43	178	416	756	2101
S2	R1	5	37	169	467	650	1807
S2	R1	6	38	144	332	638	2121
S2	R1	7	33	162	383	758	1593
S2	R1	8	35	178	400	670	1836
S2	R1	9	34	141	418	716	
S2	R1	10	33	165	400	692	
S2	R1	11	38	160	342		
S2	R2	1	37	168	339	741	2266
S2	R2	2	40	178	520	674	1715
S2	R2	3	33	162	397	592	1867
S2	R2	4	34	176	392	786	1895
S2	R2	5	36	145	478	832	1707
S2	R2	6	41	184	440	542	1931
S2	R2	7	34	157	433	622	2163
S2	R2	8	39	141	378	708	1645
S2	R2	9	34	154	412	680	
S2	R2	10	37	182	532	640	
S2	R2	11	34	162	406		
S2	R3	1	37	146	446	691	2094
S2	R3	2	37	150	428	736	1700
S2	R3	3	44	156	390	826	1996
S2	R3	4	36	176	339	756	1820
S2	R3	5	42	150	365	586	2007
S2	R3	6	36	149	434	660	1891
S2	R3	7	41	143	400	766	1946
S2	R3	8	40	170	362	730	1852
S2	R3	9	35	166	460	668	
S2	R3	10	35	145	396	632	
S2	R3	11	42	179	500		
S2	R4	1	37	192	413	800	1948
S2	R4	2	38	175	442	730	2020
S2	R4	3	37	170	417	744	1976
S2	R4	4	37	152	426	678	1884
S2	R4	5	38	195	429	740	1796
S2	R4	6	36	164	423	788	1882
S2	R4	7	45	172	495	698	1891
S2	R4	8	37	162	438	760	2000
S2	R4	9	37	180	429	716	
S2	R4	10	34	182	389	632	
S2	R4	11	40	172	407		
S2	R5	1	41	194	458	810	2158
S2	R5	2	34	178	386	772	2100
S2	R5	3	34	164	442	638	1909
S2	R5	4	34	115	436	776	1927
S2	R5	5	35	170	460	460	1952
S2	R5	6	33	165	442	788	1881
S2	R5	7	36	125	358	754	
S2	R5	8	41	145	278	794	
S2	R5	9	38	163	387	678	
S2	R5	10	38	143	382	702	
S2	R5	11	42	158	460		
S2	R6	1	33	181	454	845	2178
S2	R6	2	39	176	429	742	2051
S2	R6	3	39	143	396	680	1800
S2	R6	4	39	161	392	640	2018
S2	R6	5	41	152	428	706	1719
S2	R6	6	38	165	365	672	2020
S2	R6	7	33	135	436	684	1746
S2	R6	8	37	158	463	766	
S2	R6	9	34	144	391	640	
S2	R6	10	40	158	440	764	
S2	R6	11	34	170	360		
Mean			37.06	162.32	415.26	710.85	

Weight of supplementation 3 (S3)							
			Day 1	Day 7	Day 14	Day 21	Day 35
Supp.	Rep.	Num.	Weight 1 (g)	Weight 2 (g)	Weight 3 (g)	Weight 4 (g)	Weight 5 (g)
S3	R1	1	33	177	400	747	2028
S3	R1	2	37	163	455	744	1916
S3	R1	3	37	173	366	694	1815
S3	R1	4	38	155	368	656	1674
S3	R1	5	36	154	405	520	1991
S3	R1	6	35	158	318	660	1701
S3	R1	7	35	190	390	588	1510
S3	R1	8	40	147	272	830	1785
S3	R1	9	36	164	347	788	
S3	R1	10	42	137	346	652	
S3	R1	11	33	200	417		
S3	R2	1	37	169	426	710	2076
S3	R2	2	33	156	386	560	1881
S3	R2	3	35	157	391	566	1695
S3	R2	4	33	158	445	660	2177
S3	R2	5	36	179	445	766	1807
S3	R2	6	36	172	409	772	1883
S3	R2	7	40	163	338	780	2016
S3	R2	8	33	120	422	730	2172
S3	R2	9	36	167	376	684	
S3	R2	10	38	155	314	694	
S3	R2	11	38	139	441		
S3	R3	1	33	204	430	767	2087
S3	R3	2	35	159	485	584	2152
S3	R3	3	35	187	378	802	1990
S3	R3	4	35	186	373	710	2130
S3	R3	5	37	156	384	646	1517
S3	R3	6	39	164	410	750	1791
S3	R3	7	39	164	344	848	1578
S3	R3	8	34	151	441	724	1900
S3	R3	9	35	174	480	656	
S3	R3	10	39	154	368	670	
S3	R3	11	43	154	428		
S3	R4	1	34	141	386	684	1908
S3	R4	2	35	152	343	688	2012
S3	R4	3	33	137	359	518	1770
S3	R4	4	37	151	374	622	1707
S3	R4	5	42	148	357	654	1482
S3	R4	6	35	171	326	652	1787
S3	R4	7	35	137	349	618	1669
S3	R4	8	40	184	286	730	1719
S3	R4	9	39	126	322	628	
S3	R4	10	35	141	362	552	
S3	R4	11	38	157	277		
S3	R5	1	39	207	380	869	2163
S3	R5	2	36	180	426	832	2075
S3	R5	3	38	183	490	672	1859
S3	R5	4	34	180	471	634	1843
S3	R5	5	44	181	500	614	1793
S3	R5	6	33	130	510	640	1821
S3	R5	7	34	161	377	834	1900
S3	R5	8	42	140	460	778	1935
S3	R5	9	37	170	353	756	
S3	R5	10	33	144	374	616	
S3	R5	11	36	153	382		
S3	R6	1	36	184	318	825	1944
S3	R6	2	34	158	432	652	1934
S3	R6	3	36	137	456	682	1981
S3	R6	4	37	169	399	652	1832
S3	R6	5	37	151	387	542	1732
S3	R6	6	36	185	373	612	1730
S3	R6	7	35	154	380	754	1681
S3	R6	8	35	142	426	606	1635
S3	R6	9	38	162	378	662	
S3	R6	10	35	144	338	560	
S3	R6	11	38	148	359		
Mean			36.47	160.82	389.52	684.93	

SM: Suplementary material, C1: Base diet, C2: Base diet +50 ppm Zin bacitracin, S1: supplemented 5.63 mg/kg BW, S2: supplemented 11.0 mg/Kg BW, S3: supplemented 16.3 mg/Kg BW MCEE
