# Supplementary material for: Ethanolic extract of Morinda citrifolia improves gut microbiota, intestinal morphology, and performance without adverse effects on hematological profiles in broiler chickens
Source: Front Vet Sci. 2026 Jan 28;12:1686136. doi: 10.3389/fvets.2025.1686136 (PMC12892492; doi:10.3389/fvets.2025.1686136)
Supplement: Supplementary file 3 [file Data_Sheet_3.pdf]

SM TABLE 3: DATA OF GUT HISTOLOGY OF CHICKENS SUPPLEMENTED 5.63, 11.0 AND 16.3 mg/Kg BW MCEE DAILY

			7 days of age				14 days of age				23 days of age			
		R1	VH	CD	VW	VH/CD	VH	CD	VW	VH/CD	VH	CD	VW	VH/CD
		DUODENUM	1426.3	259.3	122.1	5.5	1913.0	255.8	146.3	7.48	1679.5	253.9	128.9	6.61
		YEYUNUM	1031.6	254.0	119.5	4.1	1174.8	257.8	144.2	4.56	1361.5	293.5	128.6	4.64
	R1	ILEUM	751.1	173.8	118.6	4.3	1013.0	208.6	118.3	4.86	885.4	201.3	109.8	4.40
		DUODENUM	1633.8	221.7	113.3	7.4	1613.3	296.3	157.0	5.44	1518.8	291.6	126.9	5.21
		YEYUNUM	893.3	155.1	114.6	5.8	831.7	207.8	131.8	4.00	1150.0	274.0	109.4	4.20
	R2	ILEUM	870.0	265.6	123.4	3.3	628.0	148.6	114.2	4.23	936.1	178.3	135.5	5.25
		DUODENUM	1436.3	170.5	121.0	8.4	1481.3	203.3	159.8	7.29	1500.9	277.4	147.1	5.41
		YEYUNUM	965.3	168.0	119.3	5.7	1118.6	223.3	111.7	5.01	1268.8	313.2	153.1	4.05
	R3	ILEUM	650.4	136.1	119.0	4.8	747.2	160.9	106.3	4.64	729.4	175.4	115.1	4.16
		DUODENUM	1395.0	201.4	124.6	6.9	1495.4	221.0	145.3	6.77	1512.1	237.6	139.5	6.36
		YEYUNUM	964.5	238.3	140.3	4.0	1042.4	211.2	145.9	4.94	1234.0	223.0	111.4	5.53
	R4	ILEUM	729.6	201.8	112.4	3.6	687.0	167.2	136.3	4.11	976.8	190.9	113.3	5.12
		DUODENUM	1316.1	169.6	125.9	7.8	1760.1	239.1	152.4	7.36	1470.7	198.2	112.7	7.42
		YEYUNUM	948.2	203.2	107.0	4.7	1139.0	218.0	115.6	5.22	1167.9	266.6	121.2	4.38
	R5	ILEUM	533.8	125.9	121.1	4.2	694.4	181.2	110.2	3.83	683.3	197.0	111.8	3.47
		DUODENUM	1543.0	222.2	138.2	6.9	1818.6	264.1	186.3	6.89	1597.0	238.6	140.0	6.69
Control		YEYUNUM	990.4	188.6	106.0	5.3	926.3	208.3	134.4	4.45	1025.4	190.6	109.4	5.38
1 (C1)	R6	ILEUM	587.2	142.5	119.1	4.1	770.9	163.1	128.8	4.73	835.1	194.6	111.9	4.29
		DUODENUM	1466.4	168.4	146.0	8.7	1552.7	242.3	156.1	6.41	1779.2	268.3	125.1	6.63
		YEYUNUM	1037.6	220.4	119.8	4.7	1104.8	217.4	116.3	5.08	1234.3	285.0	111.3	4.33
	R1	ILEUM	780.6	193.8	127.2	4.0	738.2	155.0	107.6	4.76	981.4	252.3	110.8	3.89
		DUODENUM	1534.7	244.5	128.9	6.3	1576.0	239.5	170.2	6.58	1658.6	270.7	148.4	6.13
		YEYUNUM	963.9	254.9	129.1	3.8	1062.9	248.7	119.9	4.27	1239.0	220.3	140.0	5.62
	R2	ILEUM	657.8	143.0	111.2	4.6	761.4	201.1	116.6	3.79	691.7	165.0	129.3	4.19
		DUODENUM	1411.6	266.0	130.5	5.3	1627.9	227.5	145.3	7.16	1458.7	251.8	123.7	5.79
		YEYUNUM	876.3	150.3	109.8	5.8	867.8	194.3	127.1	4.47	1024.2	256.9	121.4	3.99
	R3	ILEUM	733.8	188.0	133.9	3.9	841.1	158.6	117.2	5.30	806.3	194.4	105.8	4.15
		DUODENUM	1451.5	241.6	131.7	6.0	1887.7	319.5	136.1	5.91	1802.9	233.8	138.6	7.71
		YEYUNUM	1100.8	230.1	114.9	4.8	1208.5	202.5	149.4	5.97	1294.8	290.2	128.2	4.46
	R4	ILEUM	611.9	186.1	111.5	3.3	959.6	221.0	110.7	4.34	908.8	208.5	108.0	4.36
		DUODENUM	1376.7	254.1	131.2	5.4	1609.2	266.6	121.5	6.04	1797.2	245.8	130.5	7.31
		YEYUNUM	1093.7	256.7	137.1	4.3	1237.5	202.8	140.7	6.10	1050.4	214.6	113.6	4.89
	R5	ILEUM	841.0	154.3	108.3	5.5	693.5	186.2	108.8	3.72	850.3	175.0	114.5	4.86
		DUODENUM	1410.8	266.4	130.2	5.3	1820.6	252.3	156.0	7.22	1745.7	305.3	133.4	5.72
Control		YEYUNUM	763.3	172.8	102.8	4.4	1152.1	217.7	128.9	5.29	1213.4	302.4	124.1	4.01
2 (C2)	R6	ILEUM	720.0	225.6	131.6	3.2	798.4	165.5	121.4	4.82	768.8	182.9	115.3	4.20
		DUODENUM	1373.8	262.2	132.5	5.2	1475.1	265.7	137.2	5.55	1712.1	307.5	138.8	5.57
		YEYUNUM	992.7	201.7	108.0	4.9	974.7	206.1	129.9	4.73	1289.2	277.8	128.4	4.64
	R1	ILEUM	596.9	193.8	116.2	3.1	698.4	179.3	118.9	3.90	606.5	204.0	127.6	2.97
		DUODENUM	1220.5	209.4	118.2	5.8	1450.5	224.2	154.7	6.47	1615.9	250.0	121.5	6.46
		YEYUNUM	626.7	216.8	130.9	2.9	993.0	168.0	122.7	5.91	1242.6	208.0	115.4	5.97
	R2	ILEUM	485.5	132.5	124.4	3.7	815.7	141.4	118.1	5.77	947.1	241.4	119.3	3.92
		DUODENUM	1297.6	301.4	121.4	4.3	1654.5	261.0	134.0	6.34	1721.8	297.2	132.0	5.79
		YEYUNUM	912.5	212.9	131.9	4.3	885.5	220.5	125.1	4.02	1278.5	296.0	118.7	4.32
	R3	ILEUM	726.2	142.2	112.7	5.1	826.9	204.8	119.6	4.04	921.8	227.9	107.2	4.04
		DUODENUM	1549.0	236.6	123.3	6.5	1692.4	292.6	121.0	5.78	1807.6	281.0	129.3	6.43

		YEYUNUM	887.1	216.1	128.7	4.1	779.4	167.0	137.8	4.67	1163.6	248.1	114.5	4.69
	R4	ILEUM	597.6	218.9	132.0	2.7	644.0	143.2	106.7	4.50	864.4	161.0	119.9	5.37
		DUODENUM	1517.1	229.1	122.2	6.6	1911.2	265.0	141.8	7.21	1807.7	302.4	107.5	5.98
		YEYUNUM	1145.6	214.3	106.9	5.3	947.5	196.8	126.7	4.81	1532.0	267.9	146.8	5.72
Supple	R5	ILEUM	731.9	143.4	115.0	5.1	736.5	181.1	125.7	4.07	971.9	195.8	108.1	4.96
mentati		DUODENUM	1402.9	182.0	118.5	7.7	1645.0	264.8	121.7	6.21	1875.5	384.5	121.1	4.88
on 1		YEYUNUM	748.4	139.0	112.2	5.4	1104.9	222.6	118.1	4.96	1328.6	263.2	116.7	5.05
(S1)	R6	ILEUM	605.9	169.7	125.0	3.6	726.2	148.9	117.0	4.88	746.0	208.4	123.6	3.58
		DUODENUM	1486.2	236.5	127.3	6.3	1531.5	241.6	114.5	6.34	1807.1	383.1	130.8	4.72
		YEYUNUM	798.9	165.7	117.1	4.8	984.0	234.0	125.5	4.21	993.8	234.4	117.6	4.24
	R1	ILEUM	607.2	139.2	107.4	4.4	786.0	225.0	128.0	3.49	682.4	149.3	116.5	4.57
		DUODENUM	1447.1	173.9	114.0	8.3	1812.9	225.5	120.9	8.04	1764.9	292.4	122.2	6.04
		YEYUNUM	1023.6	226.9	113.0	4.5	832.6	189.5	117.0	4.39	1108.6	294.4	110.8	3.77
	R2	ILEUM	782.5	225.0	123.2	3.5	552.9	136.9	123.6	4.04	839.1	169.6	104.8	4.95
		DUODENUM	1310.1	282.1	115.1	4.6	1591.0	270.6	126.1	5.88	1708.0	355.0	120.4	4.81
		YEYUNUM	896.1	140.6	127.7	6.4	1271.6	252.4	111.7	5.04	1098.0	282.8	118.3	3.88
	R3	ILEUM	754.5	179.2	130.1	4.2	775.7	175.8	127.7	4.41	811.3	202.7	117.0	4.00
		DUODENUM	1286.4	215.1	119.9	6.0	1697.6	278.1	136.5	6.10	1695.3	304.1	123.0	5.57
		YEYUNUM	814.3	128.6	126.7	6.3	1222.4	236.1	113.8	5.18	1285.0	222.5	107.5	5.78
	R4	ILEUM	614.2	173.0	127.5	3.6	787.8	187.4	109.8	4.20	772.7	174.1	107.3	4.44
		DUODENUM	1448.4	233.8	142.4	6.2	1608.3	236.5	132.0	6.80	1773.0	316.8	137.8	5.60
		YEYUNUM	945.4	220.7	129.4	4.3	855.8	236.4	132.0	3.62	1038.0	244.1	122.4	4.25
Supple	R5	ILEUM	670.9	140.0	109.4	4.8	828.1	171.0	127.0	4.84	690.6	167.4	121.0	4.13
mentati		DUODENUM	1249.2	243.5	123.6	5.1	1640.7	274.5	123.6	5.98	1620.7	276.2	141.4	5.87
on 2		YEYUNUM	868.9	192.1	141.6	4.5	859.6	220.1	127.8	3.90	1371.9	257.0	128.1	5.34
(S2)	R6	ILEUM	633.1	131.7	118.1	4.8	770.6	195.2	135.5	3.95	995.4	292.4	127.0	3.40
		DUODENUM	1560.6	299.2	131.2	5.2	1703.1	278.6	111.4	6.11	1692.5	251.0	137.8	6.74
		YEYUNUM	1022.6	316.3	127.8	3.2	1129.7	242.2	118.7	4.66	1214.0	243.9	121.7	4.98
	R1	ILEUM	431.4	147.4	121.1	2.9	926.8	181.5	1069.0	5.11	612.7	136.6	107.9	4.49
		DUODENUM	1439.5	236.4	117.3	6.1	1784.9	248.4	112.9	7.19	1730.7	259.5	108.9	6.67
		YEYUNUM	877.4	167.9	125.5	5.2	1327.9	286.5	111.0	4.63	1173.3	215.8	111.1	5.44
	R2	ILEUM	557.3	126.7	119.4	4.4	910.8	221.4	107.5	4.11	810.1	169.4	125.0	4.78
		DUODENUM	1249.8	167.0	116.3	7.5	1449.4	263.0	125.3	5.51	1817.4	299.0	128.3	6.08
		YEYUNUM	826.9	181.3	105.7	4.6	973.4	201.4	123.8	4.83	1082.4	224.5	109.9	4.82
	R3	ILEUM	576.2	152.0	119.8	3.8	684.0	170.7	142.8	4.01	772.2	160.2	107.0	4.82
		DUODENUM	899.8	178.1	111.0	5.1	1732.8	235.0	183.9	7.37	1457.6	185.4	124.7	7.86
		YEYUNUM	585.7	136.6	125.1	4.3	861.8	191.6	140.8	4.50	1072.1	194.0	117.3	5.53
	R4	ILEUM	396.4	135.0	108.6	2.9	821.4	149.3	109.4	5.50	729.0	177.0	119.7	4.12
		DUODENUM	1352.8	341.0	125.5	4.0	1582.0	252.7	114.3	6.26	1537.4	288.7	117.3	5.33
		YEYUNUM	986.7	179.8	116.3	5.5	1339.0	200.5	122.1	6.68	1253.3	204.4	103.2	6.13
Supple	R5	ILEUM	435.6	155.8	119.2	2.8	968.3	162.4	116.3	5.96	763.9	193.3	116.1	3.95
mentati		DUODENUM	1395.4	197.7	132.3	7.1	2013.9	336.2	123.6	5.99	1660.0	264.0	132.8	6.29
on 3		YEYUNUM	859.6	196.4	120.1	4.4	1184.9	256.1	120.0	4.63	1066.0	137.9	111.6	7.73
(S3)	R6	ILEUM	508.4	145.7	124.8	3.5	840.6	168.9	130.9	4.98	798.6	167.1	114.2	4.78

SM: supplemenary material, VH: Villus height, CD: Crypt dept, VW: villus width
