# Supplementary material for: Ethanolic extract of Morinda citrifolia improves gut microbiota, intestinal morphology, and performance without adverse effects on hematological profiles in broiler chickens
Source: Front Vet Sci. 2026 Jan 28;12:1686136. doi: 10.3389/fvets.2025.1686136 (PMC12892492; doi:10.3389/fvets.2025.1686136)
Supplement: Supplementary file 4 [file Data_Sheet_4.pdf]

**SM TABLE 4: DATA COUNTING OF COLONY FORMING UNITS IN ILEUM CONTENT OF CHICKENS SUPPLEMENTED 5.63, 11.0, 16.30 MG/KG BW DAILY OF *MORINDA CITRIFOLIA* ETHANOLIC EXTRACT**

| ***ESCHERICHIA COLI* - MC** | | | | | | | | | |
|---|---|---|---|---|---|---|---|---|---|
| **10 fold dilutions** | | | | | | | | | |
| **samp** | **Suppl/replica** | **-1** | **-2** | **-3** | **-4** | **-4** | **-5** | **-5** | **N° CFU/g** |
| 1 | C1R1 | M | 238 | 31 | 3 | 5 | 2 | 1 | 150000 |
| 2 | C1R2 | M | M | 160 | 20 | 35 | 3 | 4 | 160000 |
| 3 | C1R3 | M | 160 | 50 | 15 | 15 | 18 | 10 | 150000 |
| 4 | C2R1 | M | M | M | 34 | 72 | 2 | 72 | 530000 |
| 5 | C2R2 | M | M | 384 | 46 | 50 | 21 | 10 | 384000 |
| 6 | C2R3 | M | M | M | 128 | 56 | 56 | 17 | 920000 |
| 7 | S1R1 | M | M | 110 | 0 | 0 | 0 | 0 | 11000 |
| 8 | S1R2 | M | M | M | 80 | 84 | 8' | 58 | 12100 |
| 9 | S1R3 | M | 132 | 45 | 22 | 28 | 5 | 6 | 13200 |
| 10 | S2R1 | M | 760 | 15 | 5 | 3 | 0 | 1 | 76000 |
| 11 | S2R2 | M | 572 | 360 | 13 | 4 | 0 | 0 | 57200 |
| 12 | S2R3 | M | M | 744 | 37 | 21 | 2 | 5 | 66000 |
| 13 | S3R1 | 880 | 8 | 4 | 1 | 1 | 0 | 0 | 8800 |
| 14 | S3R2 | 160 | 5 | 2 | 1 | 0 | 0 | 1 | 1600 |
| 15 | S3R3 | 828 | 210 | 10 | 2 | 4 | 1 | 0 | 8280 |

| ***STAPHYLOCOCCUS AUREUS* - SM** | | | | | | | | | |
|---|---|---|---|---|---|---|---|---|---|
| **10 fold dilutions** | | | | | | | | | |
| **samp** | **Suppl/replica** | **-1** | **-2** | **-3** | **-4** | **-4** | **-5** | **-5** | **N° CFU/g** |
| 1 | C1R1 | M | 96 | 54 | 15 | C | 17 | 13 | 1500000 |
| 2 | C1R2 | M | M | M | 74 | C | 64 | 41 | 740000 |
| 3 | C1R3 | M | M | M | 66 | 46 | 3 | 22 | 560000 |
| 4 | C2R1 | M | M | M | 32 | 27 | 15 | 13 | 295000 |
| 5 | C2R2 | M | M | 248 | 60 | 63 | 43 | 33 | 248000 |
| 6 | C2R3 | M | M | 43 | 46 | 43 | 32 | 24 | 288000 |
| 7 | S1R1 | M | M | 328 | C | 16 | 11 | 7 | 328000 |
| 8 | S1R2 | M | M | 180 | 26 | 27 | 7 | 10 | 180000 |
| 9 | S1R3 | M | M | 120 | 38 | 35 | 13 | 10 | 120000 |
| 10 | S2R1 | M | M | M | 41 | 14 | 6 | 5 | 410000 |
| 11 | S2R2 | M | M | M | 70 | 33 | 7 | C | 700000 |
| 12 | S2R3 | M | M | M | 100 | 136 | 44 | 40 | 555000 |
| 13 | S3R1 | M | M | 31 | 34 | 26 | 14 | 4 | 31000 |
| 14 | S3R2 | M | M | 176 | 109 | 67 | 21 | 10 | 176000 |
| 15 | S3R3 | M | M | 86 | 56 | 32 | 15 | 3 | 86000 |

| ***LACTOBACILLUS SP* - MRS** | | | | | | | | | |
|---|---|---|---|---|---|---|---|---|---|
| **10 fold dilutions** | | | | | | | | | |
| **samp** | **Suppl/replica** | **-1** | **-2** | **-3** | **-4** | **-4** | **-5** | **-5** | **N° CFU/g** |
| 1 | C1R1 | M | M | M | 148 | C | C | C | 1480000 |
| 2 | C1R2 | M | M | M | 212 | 356 | 168 | 12 | 2850000 |
| 3 | C1R3 | M | M | M | 368 | 268 | 41 | 63 | 3180000 |

| | | | | | | | | | |
|---|---|---|---|---|---|---|---|---|---|
| 4 | C2R1 | M | M | M | 172 | 120 | 18 | 240 | 1460000 |
| 5 | C2R2 | M | M | M | 320 | 280 | 110 | 75 | 3000000 |
| 6 | C2R3 | M | M | 384 | 176 | 172 | 34 | 56 | 1740000 |
| 7 | S1R1 | M | 270 | 32 | 1 | 7 | 1 | 0 | 27000 |
| 8 | S1R2 | M | M | 436 | 184 | 32 | 38 | 26 | 436000 |
| 9 | S1R3 | M | M | 186 | 16 | 18 | 7 | 82 | 170000 |
| 10 | S2R1 | 79 | 12 | 15 | 2 | 1 | 0 | 0 | 15000 |
| 11 | S2R2 | 10 | 12 | 3 | 3 | 13 | 1 | 0 | 50000 |
| 12 | S2R3 | M | M | 768 | 36 | 7 | 2 | 0 | 768000 |
| 13 | S3R1 | M | M | 144 | 132 | 96 | 60 | 52 | 144000 |
| 14 | S3R2 | M | M | 240 | 10 | 15 | 7 | 12 | 240000 |
| 15 | S3R3 | M | M | 436 | 37 | 50 | 21 | 12 | 436000 |

SM: supplementary material, MC: MC Conkey agar, MA: manitol agar, MRS: de Man, Rogosa and Sharpe agar, C1: base diet, C2: Base diet +50 ppm Zin bacitracin, S1: supplemented 5.63 mg/kg BW, S2: supplemented 11.0 mg/Kg BW, S3: supplemented 16.3 mg/Kg BW MCEE, C: contaminated, CFU/g: Colony forming unit per gram of sample
