# Supplementary material for: Ethanolic extract of Morinda citrifolia improves gut microbiota, intestinal morphology, and performance without adverse effects on hematological profiles in broiler chickens
Source: Front Vet Sci. 2026 Jan 28;12:1686136. doi: 10.3389/fvets.2025.1686136 (PMC12892492; doi:10.3389/fvets.2025.1686136)
Supplement: Supplementary file 5 [file Data_Sheet_5.pdf]

SM TABLE 5: R-STATISTICAL ANALYSIS PROCEDURES OF FEED INTAKE AND GROWTH PERFORMANCE OF CHICKENS SUPPLEMENTED 5.63, 11.0, 16.30 MG/KG BW DAILY OF MORINDA CITRIFOLIA ETHANOLIC EXTRACT.

```
#############################
## II. Consumo de Alimento #
#############################
library(readxl)
library(dplyr)
library(car)
library(nortest)
library(e1071)
library(lmtest)
library(emmeans)

# Lectura de datos
Datos <- read_excel("01 - Parámetros Productivos.xlsx",
          sheet = "Consumo de Alimento",
          range = "X4:AC34")
Datos$TRATA <- factor(Datos$TRATA)
head(Datos)

## 2.1. Etapa de Inicio

# Resumen descriptivo
Datos %>%
  group_by(TRATA) %>%
  summarise(n = n(),
       Media = mean(INI),
       Minimo = min(INI),
```

```
            Maximo = max(INI),
            SD = sd(INI),
            CV = 100*SD/Media,
            MAD = median(abs(INI-median(INI))),
            Z = max(abs((INI-Media)/SD))) %>%
  data.frame()

# Estimar el modelo
mod.caini <- lm(INI ~ TRATA, data = Datos)
anova(mod.caini)

# Resumen del modelo
summary(aov(mod.caini))

# Coeficiente de variacion
SD <- sqrt(deviance(mod.caini)/df.residual(mod.caini))
100*SD/mean(predict(mod.caini))

# Coeficiente de determinación
summary(mod.caini)

# Analisis de los residuales estandarizados
data.frame(res.stand = rstandard(mod.caini)) %>%
  summarise(Minimo = min(res.stand),
            Maximo = max(res.stand),
            SD = sd(res.stand),
            valorp_Shapiro = shapiro.test(res.stand)$p.value,
            valorp_Adarlin = ad.test(res.stand)$p.value,
```

```
          valorp_KS = ks.test(res.stand,"pnorm")$p.value,
          Asimetria = skewness(res.stand),
          Curtosis = kurtosis(res.stand),
          valorp_ncVtest = ncvTest(mod.caini)$p,
          valorp_bptest = bptest(mod.caini)$p.value) %>%
  data.frame() %>%
  t()

# Tamaño del efecto
HSD.test(y = Datos$INI,
        trt = Datos$TRATA,
        DFerror = df.residual(mod.caini),
        MSerror = deviance(mod.caini)/df.residual(mod.caini),
        alpha = 0.05,
        group = FALSE)$comparison %>%
  mutate(CME = anova(mod.caini)["Residuals", "Mean Sq"],
        Cohen = difference / CME,
        across(where(is.numeric), ~ round(., 2))) %>%
  dplyr::select(difference, Cohen, pvalue)

## 2.2. Etapa de Crecimiento

# Resumen descriptivo
Datos %>%
  group_by(TRATA) %>%
  summarise(n = n(),
          Media = mean(CREC),
          Minimo = min(CREC),
```

```
          Maximo = max(CREC),
          SD = sd(CREC),
          CV = 100*SD/Media,
          MAD = median(abs(CREC-median(CREC))),
          Z = max(abs((CREC-Media)/SD))) %>%
  data.frame()

# Estimar el modelo
mod.cacrec <- lm(CREC ~ TRATA, data = Datos)

# Resumen del modelo
summary(aov(mod.cacrec))

# Coeficiente de variacion
SD <- sqrt(deviance(mod.cacrec)/df.residual(mod.cacrec))
100*SD/mean(predict(mod.cacrec))

# Coeficiente de determinación
summary(mod.cacrec)

# Analisis de los residuales estandarizados
data.frame(res.stand = rstandard(mod.cacrec)) %>%
  summarise(Minimo = min(res.stand),
          Maximo = max(res.stand),
          SD = sd(res.stand),
          valorp_Shapiro = shapiro.test(res.stand)$p.value,
          valorp_Adarlin = ad.test(res.stand)$p.value,
          valorp_KS = ks.test(res.stand,"pnorm")$p.value,
```

```
          Asimetria = skewness(res.stand),
          Curtosis = kurtosis(res.stand),
          valorp_ncVtest = ncvTest(mod.cacrec)$p,
          valorp_bptest = bptest(mod.cacrec)$p.value) %>%
  data.frame() %>%
  t()

# Identificar valores atipicos
subset(rstandard(mod.cacrec),
       rstandard(mod.cacrec) > 2.5| rstandard(mod.cacrec) < - 2.5)

# Tamaño del efecto
HSD.test(y = Datos$CREC,
         trt = Datos$TRATA,
         DFerror = df.residual(mod.cacrec),
         MSerror = deviance(mod.cacrec)/df.residual(mod.cacrec),
         alpha = 0.05,
         group = FALSE)$comparison %>%
  mutate(CME = anova(mod.cacrec)["Residuals", "Mean Sq"],
         Cohen = difference / CME,
         across(where(is.numeric), ~ round(., 2))) %>%
  dplyr::select(difference, Cohen, pvalue)

## 2.3. Etapa de Engorde

# Resumen descriptivo
Datos %>%
  group_by(TRATA) %>%
```

```
  summarise(n = n(),
            Media = mean(ENG),
            Minimo = min(ENG),
            Maximo = max(ENG),
            SD = sd(ENG),
            CV = 100*SD/Media,
            MAD = median(abs(ENG-median(ENG))),
            Z = max(abs((ENG-Media)/SD))) %>%
  data.frame()

# Estimar el modelo
mod.caeng <- lm(ENG ~ TRATA, data = Datos)

# Resumen del modelo
summary(aov(mod.caeng))

# Coeficiente de variacion
SD <- sqrt(deviance(mod.caeng)/df.residual(mod.caeng))
100*SD/mean(predict(mod.caeng))

# Coeficiente de determinación
summary(mod.caeng)

# Analisis de los residuales estandarizados
data.frame(res.stand = rstandard(mod.caeng)) %>%
  summarise(Minimo = min(res.stand),
            Maximo = max(res.stand),
            SD = sd(res.stand),
```

```
        valorp_Shapiro = shapiro.test(res.stand)$p.value,
        valorp_Adarlin = ad.test(res.stand)$p.value,
        valorp_KS = ks.test(res.stand,"pnorm")$p.value,
        Asimetria = skewness(res.stand),
        Curtosis = kurtosis(res.stand),
        valorp_ncVtest = ncvTest(mod.caeng)$p,
        valorp_bptest = bptest(mod.caeng)$p.value) %>%
  data.frame() %>%
  t()

# Identificar valores atipicos
subset(rstandard(mod.caeng),
      rstandard(mod.caeng) > 2.5 | rstandard(mod.caeng) < -2.5)

# Tamaño del efecto
HSD.test(y = Datos$ENG,
      trt = Datos$TRATA,
      DFerror = df.residual(mod.caeng),
      MSerror = deviance(mod.caeng)/df.residual(mod.caeng),
      alpha = 0.05,
      group = FALSE)$comparison %>%
  mutate(CME = anova(mod.caeng)["Residuals", "Mean Sq"],
      Cohen = difference / CME,
      across(where(is.numeric), ~ round(., 2))) %>%
  dplyr::select(difference, Cohen, pvalue)

## 2.4. Etapa Total
```

```
# Resumen descriptivo
Datos %>%
  group_by(TRATA) %>%
  summarise(n = n(),
            Media = mean(TOT),
            Minimo = min(TOT),
            Maximo = max(TOT),
            SD = sd(TOT),
            CV = 100*SD/Media,
            MAD = median(abs(TOT-median(TOT))),
            Z = max(abs((TOT-Media)/SD))) %>%
  data.frame()

# Estimar el modelo
mod.catot <- lm(TOT ~ TRATA, data = Datos)

# Resumen del modelo
summary(aov(mod.catot))

# Coeficiente de variacion
SD <- sqrt(deviance(mod.catot)/df.residual(mod.catot))
100*SD/mean(predict(mod.catot))

# Coeficiente de determinación
summary(mod.catot)

# Analisis de los residuales estandarizados
data.frame(res.stand = rstandard(mod.catot)) %>%
```

```
  summarise(Minimo = min(res.stand),
            Maximo = max(res.stand),
            SD = sd(res.stand),
            valorp_Shapiro = shapiro.test(res.stand)$p.value,
            valorp_Adarlin = ad.test(res.stand)$p.value,
            valorp_KS = ks.test(res.stand,"pnorm")$p.value,
            Asimetria = skewness(res.stand),
            Curtosis = kurtosis(res.stand),
            valorp_ncVtest = ncvTest(mod.catot)$p,
            valorp_bptest = bptest(mod.catot)$p.value) %>%
  data.frame() %>%
  t()

# Identificar valores atipicos
subset(rstandard(mod.catot),
       rstandard(mod.catot) > 2.5 | rstandard(mod.catot) < -2.5)

# Tamaño del efecto
HSD.test(y = Datos$TOT,
         trt = Datos$TRATA,
         DFerror = df.residual(mod.catot),
         MSerror = deviance(mod.catot)/df.residual(mod.catot),
         alpha = 0.05,
         group = FALSE)$comparison %>%
  mutate(CME = anova(mod.catot)["Residuals", "Mean Sq"],
         Cohen = difference / CME,
         across(where(is.numeric), ~ round(., 2))) %>%
  dplyr::select(difference, Cohen, pvalue)
```

```
##############
## III. Peso #
##############

# Lectura de datos
Datos <- read_excel("01 - Parámetros Productivos.xlsx",
                   sheet = "Peso",
                   range = "M5:R35")
Datos$TRATA <- factor(Datos$TRATA)
head(Datos)

## 3.1. Etapa de Recepción

# Resumen descriptivo
Datos %>%
  group_by(TRATA) %>%
  summarise(n = n(),
            Media = mean(REC),
            Minimo = min(REC),
            Maximo = max(REC),
            SD = sd(REC),
            CV = 100*SD/Media,
            MAD = median(abs(REC-median(REC))),
            Z = max(abs((REC-Media)/SD))) %>%
  data.frame()

# Estimar el modelo
```

```r
mod.wrec <- lm(REC ~ TRATA, data = Datos)

# Resumen del modelo
summary(aov(mod.wrec))

# Coeficiente de variacion
SD <- sqrt(deviance(mod.wrec)/df.residual(mod.wrec))
100*SD/mean(predict(mod.wrec))

# Coeficiente de determinación
summary(mod.wrec)

# Analisis de los residuales estandarizados
data.frame(res.stand = rstandard(mod.wrec)) %>%
  summarise(Minimo = min(res.stand),
            Maximo = max(res.stand),
            SD = sd(res.stand),
            valorp_Shapiro = shapiro.test(res.stand)$p.value,
            valorp_Adarlin = ad.test(res.stand)$p.value,
            valorp_KS = ks.test(res.stand,"pnorm")$p.value,
            Asimetria = skewness(res.stand),
            Curtosis = kurtosis(res.stand),
            valorp_ncVtest = ncvTest(mod.wrec)$p,
            valorp_bptest = bptest(mod.wrec)$p.value) %>%
  data.frame() %>%
  t()

## 3.2. Etapa de Inicio
```

```
# Resumen descriptivo
Datos %>%
  group_by(TRATA) %>%
  summarise(n = n(),
            Media = mean(INI),
            Minimo = min(INI),
            Maximo = max(INI),
            SD = sd(INI),
            CV = 100*SD/Media,
            MAD = median(abs(INI-median(INI))),
            Z = max(abs((INI-Media)/SD))) %>%
  data.frame()

# Estimar el modelo
mod.wini <- lm(INI ~ TRATA, data = Datos)

# Resumen del modelo
summary(aov(mod.wini))

# Coeficiente de variacion
SD <- sqrt(deviance(mod.wini)/df.residual(mod.wini))
100*SD/mean(predict(mod.wini))

# Coeficiente de determinación
summary(mod.wini)

# Analisis de los residuales estandarizados
```

```
data.frame(res.stand = rstandard(mod.wini)) %>%
  summarise(Minimo = min(res.stand),
            Maximo = max(res.stand),
            SD = sd(res.stand),
            valorp_Shapiro = shapiro.test(res.stand)$p.value,
            valorp_Adarlin = ad.test(res.stand)$p.value,
            valorp_KS = ks.test(res.stand,"pnorm")$p.value,
            Asimetria = skewness(res.stand),
            Curtosis = kurtosis(res.stand),
            valorp_ncVtest = ncvTest(mod.wini)$p,
            valorp_bptest = bptest(mod.wini)$p.value) %>%
  data.frame() %>%
  t()

# Identificar valores atipicos
subset(rstandard(mod.wini),
       rstandard(mod.wini) > 2.5 | rstandard(mod.wini) < -2.5)

# Tamaño del efecto
HSD.test(y = Datos$INI,
         trt = Datos$TRATA,
         DFerror = df.residual(mod.wini),
         MSerror = deviance(mod.wini)/df.residual(mod.wini),
         alpha = 0.05,
         group = FALSE)$comparison %>%
  mutate(CME = anova(mod.wini)["Residuals", "Mean Sq"],
         Cohen = difference / CME,
         across(where(is.numeric), ~ round(., 2))) %>%
```

```
  dplyr::select(difference, Cohen, pvalue)

## 3.3. Etapa de Crecimiento

# Resumen descriptivo
Datos %>%
  group_by(TRATA) %>%
  summarise(n = n(),
            Media = mean(CREC),
            Minimo = min(CREC),
            Maximo = max(CREC),
            SD = sd(CREC),
            CV = 100*SD/Media,
            MAD = median(abs(CREC-median(CREC))),
            Z = max(abs((CREC-Media)/SD))) %>%
  data.frame()

# Estimar el modelo
mod.wcrec <- lm(CREC ~ TRATA, data = Datos)

# Resumen del modelo
summary(aov(mod.wcrec))

# Coeficiente de variacion
SD <- sqrt(deviance(mod.wcrec)/df.residual(mod.wcrec))
100*SD/mean(predict(mod.wcrec))

# Coeficiente de determinación
```

```
summary(mod.wcrec)

# Analisis de los residuales estandarizados
data.frame(res.stand = rstandard(mod.wcrec)) %>%
  summarise(Minimo = min(res.stand),
            Maximo = max(res.stand),
            SD = sd(res.stand),
            valorp_Shapiro = shapiro.test(res.stand)$p.value,
            valorp_Adarlin = ad.test(res.stand)$p.value,
            valorp_KS = ks.test(res.stand,"pnorm")$p.value,
            Asimetria = skewness(res.stand),
            Curtosis = kurtosis(res.stand),
            valorp_ncVtest = ncvTest(mod.wcrec)$p,
            valorp_bptest = bptest(mod.wcrec)$p.value) %>%
  data.frame() %>%
  t()

# Identificar valores atipicos
subset(rstandard(mod.wcrec),
       rstandard(mod.wcrec) > 2.5 | rstandard(mod.wcrec) < -2.5)

# Tamaño del efecto
HSD.test(y = Datos$CREC,
         trt = Datos$TRATA,
         DFerror = df.residual(mod.wcrec),
         MSerror = deviance(mod.wcrec)/df.residual(mod.wcrec),
         alpha = 0.05,
         group = FALSE)$comparison %>%
```

```
  mutate(CME = anova(mod.wcrec)["Residuals", "Mean Sq"],
         Cohen = difference / CME,
         across(where(is.numeric), ~ round(., 2))) %>%
  dplyr::select(difference, Cohen, pvalue)

## 3.4. Etapa de Engorde

# Resumen descriptivo
Datos %>%
  group_by(TRATA) %>%
  summarise(n = n(),
            Media = mean(ENG),
            Minimo = min(ENG),
            Maximo = max(ENG),
            SD = sd(ENG),
            CV = 100*SD/Media,
            MAD = median(abs(ENG-median(ENG))),
            Z = max(abs((ENG-Media)/SD))) %>%
  data.frame()

# Estimar el modelo
mod.weng <- lm(ENG ~ TRATA, data = Datos)

# Resumen del modelo
summary(aov(mod.weng))

# Coeficiente de variacion
SD <- sqrt(deviance(mod.weng)/df.residual(mod.weng))
```

```
100*SD/mean(predict(mod.weng))

# Coeficiente de determinación
summary(mod.weng)

# Analisis de los residuales estandarizados
data.frame(res.stand = rstandard(mod.weng)) %>%
  summarise(Minimo = min(res.stand),
            Maximo = max(res.stand),
            SD = sd(res.stand),
            valorp_Shapiro = shapiro.test(res.stand)$p.value,
            valorp_Adarlin = ad.test(res.stand)$p.value,
            valorp_KS = ks.test(res.stand,"pnorm")$p.value,
            Asimetria = skewness(res.stand),
            Curtosis = kurtosis(res.stand),
            valorp_ncVtest = ncvTest(mod.weng)$p,
            valorp_bptest = bptest(mod.weng)$p.value) %>%
  data.frame() %>%
  t()

# Identificar valores atipicos
subset(rstandard(mod.weng),
       rstandard(mod.weng) > 2.5 | rstandard(mod.weng) < -2.5)

## Comparaciones multiples
SNK.test(y = Datos$ENG,
         trt = Datos$TRATA,
         DFerror = df.residual(mod.weng),
```

```
        MSerror = deviance(mod.weng)/df.residual(mod.weng),
        alpha = 0.05,
        group = FALSE)$comparison %>%
  mutate(CME = anova(mod.weng)["Residuals", "Mean Sq"],
         Cohen = difference / CME,
         across(where(is.numeric), ~ round(., 2))) %>%
  dplyr::select(difference, Cohen, pvalue)

##########################
## IV. Ganancia de Peso #
##########################

# Lectura de datos
Datos <- read_excel("01 - Parámetros Productivos.xlsx",
                    sheet = "Peso",
                    range = "S5:X35")
Datos$TRATA <- factor(Datos$TRATA)
head(Datos)

## 4.1. Etapa de Inicio

# Resumen descriptivo
Datos %>%
  group_by(TRATA) %>%
  summarise(n = n(),
            Media = mean(INI),
            Minimo = min(INI),
            Maximo = max(INI),
```

```
          SD = sd(INI),
          CV = 100*SD/Media,
          MAD = median(abs(INI-median(INI))),
          Z = max(abs((INI-Media)/SD))) %>%
  data.frame()

# Estimar el modelo
mod.gpini <- lm(INI ~ TRATA, data = Datos)

# Resumen del modelo
summary(aov(mod.gpini))

# Coeficiente de variacion
SD <- sqrt(deviance(mod.gpini)/df.residual(mod.gpini))
100*SD/mean(predict(mod.gpini))

# Coeficiente de determinación
summary(mod.gpini)

# Analisis de los residuales estandarizados
data.frame(res.stand = rstandard(mod.gpini)) %>%
  summarise(Minimo = min(res.stand),
          Maximo = max(res.stand),
          SD = sd(res.stand),
          valorp_Shapiro = shapiro.test(res.stand)$p.value,
          valorp_Adarlin = ad.test(res.stand)$p.value,
          valorp_KS = ks.test(res.stand,"pnorm")$p.value,
          Asimetria = skewness(res.stand),
```

```
          Curtosis = kurtosis(res.stand),
          valorp_ncVtest = ncvTest(mod.gpini)$p,
          valorp_bptest = bptest(mod.gpini)$p.value) %>%
  data.frame() %>%
  t()

## Comparaciones multiples
HSD.test(y = Datos$INI,
        trt = Datos$TRATA,
        DFerror = df.residual(mod.gpini),
        MSerror = deviance(mod.gpini)/df.residual(mod.gpini),
        alpha = 0.05,
        group = FALSE)$comparison %>%
  mutate(CME = anova(mod.gpini)["Residuals", "Mean Sq"],
        Cohen = difference / CME,
        across(where(is.numeric), ~ round(., 2))) %>%
  dplyr::select(difference, Cohen, pvalue)

## 4.2. Etapa de Crecimiento

# Resumen descriptivo
Datos %>%
  group_by(TRATA) %>%
  summarise(n = n(),
          Media = mean(CREC),
          Minimo = min(CREC),
          Maximo = max(CREC),
          SD = sd(CREC),
```

```
          CV = 100*SD/Media,
          MAD = median(abs(CREC-median(CREC))),
          Z = max(abs((CREC-Media)/SD))) %>%
  data.frame()

# Estimar el modelo
mod.gpcrec <- lm(CREC ~ TRATA, data = Datos)

# Resumen del modelo
summary(aov(mod.gpcrec))

# Coeficiente de variacion
SD <- sqrt(deviance(mod.gpcrec)/df.residual(mod.gpcrec))
100*SD/mean(predict(mod.gpcrec))

# Coeficiente de determinación
summary(mod.gpcrec)

# Analisis de los residuales estandarizados
data.frame(res.stand = rstandard(mod.gpcrec)) %>%
  summarise(Minimo = min(res.stand),
          Maximo = max(res.stand),
          SD = sd(res.stand),
          valorp_Shapiro = shapiro.test(res.stand)$p.value,
          valorp_Adarlin = ad.test(res.stand)$p.value,
          valorp_KS = ks.test(res.stand,"pnorm")$p.value,
          Asimetria = skewness(res.stand),
          Curtosis = kurtosis(res.stand),
```

```
          valorp_ncVtest = ncvTest(mod.gpcrec)$p,
          valorp_bptest = bptest(mod.gpcrec)$p.value) %>%
  data.frame() %>%
  t()

## Comparaciones multiples
HSD.test(y = Datos$CREC,
        trt = Datos$TRATA,
        DFerror = df.residual(mod.gpcrec),
        MSerror = deviance(mod.gpcrec)/df.residual(mod.gpcrec),
        alpha = 0.05,
        group = FALSE)$comparison %>%
  mutate(CME = anova(mod.gpcrec)["Residuals", "Mean Sq"],
        Cohen = difference / CME,
        across(where(is.numeric), ~ round(., 2))) %>%
  dplyr::select(difference, Cohen, pvalue)

## 4.3. Etapa de Engorde

# Resumen descriptivo
Datos %>%
  group_by(TRATA) %>%
  summarise(n = n(),
          Media = mean(ENG),
          Minimo = min(ENG),
          Maximo = max(ENG),
          SD = sd(ENG),
          CV = 100*SD/Media,
```

```
          MAD = median(abs(ENG-median(ENG))),
          Z = max(abs((ENG-Media)/SD))) %>%
  data.frame()

# Estimar el modelo
mod.gpeng <- lm(ENG ~ TRATA, data = Datos)

# Resumen del modelo
summary(aov(mod.gpeng))

# Coeficiente de variacion
SD <- sqrt(deviance(mod.gpeng)/df.residual(mod.gpeng))
100*SD/mean(predict(mod.gpeng))

# Coeficiente de determinación
summary(mod.gpeng)

# Analisis de los residuales estandarizados
data.frame(res.stand = rstandard(mod.gpeng)) %>%
  summarise(Minimo = min(res.stand),
          Maximo = max(res.stand),
          SD = sd(res.stand),
          valorp_Shapiro = shapiro.test(res.stand)$p.value,
          valorp_Adarlin = ad.test(res.stand)$p.value,
          valorp_KS = ks.test(res.stand,"pnorm")$p.value,
          Asimetria = skewness(res.stand),
          Curtosis = kurtosis(res.stand),
          valorp_ncVtest = ncvTest(mod.gpeng)$p,
```

```
      valorp_bptest = bptest(mod.gpeng)$p.value) %>%
  data.frame() %>%
  t()

## Comparaciones multiples
HSD.test(y = Datos$ENG,
     trt = Datos$TRATA,
     DFerror = df.residual(mod.gpeng),
     MSerror = deviance(mod.gpeng)/df.residual(mod.gpeng),
     alpha = 0.05,
     group = FALSE)$comparison %>%
  mutate(CME = anova(mod.gpeng)["Residuals", "Mean Sq"],
     Cohen = difference / CME,
     across(where(is.numeric), ~ round(., 2))) %>%
  dplyr::select(difference, Cohen, pvalue)

## 4.4. Etapa Total

# Resumen descriptivo
Datos %>%
  group_by(TRATA) %>%
  summarise(n = n(),
      Media = mean(TOT),
      Minimo = min(TOT),
      Maximo = max(TOT),
      SD = sd(TOT),
      CV = 100*SD/Media,
      MAD = median(abs(TOT-median(TOT))),
```

```
          Z = max(abs((TOT-Media)/SD))) %>%
  data.frame()

# Estimar el modelo
mod.gptot <- lm(TOT ~ TRATA, data = Datos)

# Resumen del modelo
summary(aov(mod.gptot))

# Coeficiente de variacion
SD <- sqrt(deviance(mod.gptot)/df.residual(mod.gptot))
100*SD/mean(predict(mod.gptot))

# Coeficiente de determinación
summary(mod.gptot)

# Analisis de los residuales estandarizados
data.frame(res.stand = rstandard(mod.gptot)) %>%
  summarise(Minimo = min(res.stand),
          Maximo = max(res.stand),
          SD = sd(res.stand),
          valorp_Shapiro = shapiro.test(res.stand)$p.value,
          valorp_Adarlin = ad.test(res.stand)$p.value,
          valorp_KS = ks.test(res.stand,"pnorm")$p.value,
          Asimetria = skewness(res.stand),
          Curtosis = kurtosis(res.stand),
          valorp_ncVtest = ncvTest(mod.gptot)$p,
          valorp_bptest = bptest(mod.gptot)$p.value) %>%
```

```
  data.frame() %>%
  t()

## Comparaciones multiples
HSD.test(y = Datos$TOT,
         trt = Datos$TRATA,
         DFerror = df.residual(mod.gptot),
         MSerror = deviance(mod.gptot)/df.residual(mod.gptot),
         alpha = 0.05,
         group = FALSE)$comparison %>%
  mutate(CME = anova(mod.gptot)["Residuals", "Mean Sq"],
         Cohen = difference / CME,
         across(where(is.numeric), ~ round(., 2))) %>%
  dplyr::select(difference, Cohen, pvalue)

################################
## V. Conversión Alimenticia #
################################

# Lectura de datos
choose.dir()
Datos <- read_excel("01 - Parámetros Productivos.xlsx",
                    sheet = "Peso",
                    range = "Y5:AD35")
Datos$TRATA <- factor(Datos$TRATA)

## 4.1. Etapa de Inicio
```

```
# Resumen descriptivo
Datos %>%
  group_by(TRATA) %>%
  summarise(n = n(),
            Media = mean(INI),
            Minimo = min(INI),
            Maximo = max(INI),
            SD = sd(INI),
            CV = 100*SD/Media,
            MAD = median(abs(INI-median(INI))),
            Z = max(abs((INI-Media)/SD))) %>%
  data.frame()

# Estimar el modelo
mod.caini <- lm(INI ~ TRATA, data = Datos)

# Resumen del modelo
summary(aov(mod.caini))

# Coeficiente de variacion
SD <- sqrt(deviance(mod.caini)/df.residual(mod.caini))
100*SD/mean(predict(mod.caini))

# Coeficiente de determinación
summary(mod.caini)

# Analisis de los residuales estandarizados
data.frame(res.stand = rstandard(mod.caini)) %>%
```

```
  summarise(Minimo = min(res.stand),
            Maximo = max(res.stand),
            SD = sd(res.stand),
            valorp_Shapiro = shapiro.test(res.stand)$p.value,
            valorp_Adarlin = ad.test(res.stand)$p.value,
            valorp_KS = ks.test(res.stand,"pnorm")$p.value,
            Asimetria = skewness(res.stand),
            Curtosis = kurtosis(res.stand),
            valorp_ncVtest = ncvTest(mod.caini)$p,
            valorp_bptest = bptest(mod.caini)$p.value) %>%
  data.frame() %>%
  t()

## Comparaciones multiples
HSD.test(y = Datos$INI,
         trt = Datos$TRATA,
         DFerror = df.residual(mod.caini),
         MSerror = deviance(mod.caini)/df.residual(mod.caini),
         alpha = 0.05,
         group = FALSE)$comparison %>%
  mutate(CME = anova(mod.caini)["Residuals", "Mean Sq"],
         Cohen = difference / CME,
         across(where(is.numeric), ~ round(., 2))) %>%
  dplyr::select(difference, Cohen, pvalue)

## 4.2. Etapa de Crecimiento

# Resumen descriptivo
```

```
Datos %>%
  group_by(TRATA) %>%
  summarise(n = n(),
            Media = mean(CREC),
            Minimo = min(CREC),
            Maximo = max(CREC),
            SD = sd(CREC),
            CV = 100*SD/Media,
            MAD = median(abs(CREC-median(CREC))),
            Z = max(abs((CREC-Media)/SD))) %>%
  data.frame()

# Estimar el modelo
mod.cacrec <- lm(CREC ~ TRATA, data = Datos)

# Resumen del modelo
summary(aov(mod.cacrec))

# Coeficiente de variacion
SD <- sqrt(deviance(mod.cacrec)/df.residual(mod.cacrec))
100*SD/mean(predict(mod.cacrec))

# Coeficiente de determinación
summary(mod.cacrec)

# Analisis de los residuales estandarizados
data.frame(res.stand = rstandard(mod.cacrec)) %>%
  summarise(Minimo = min(res.stand),
```

```
          Maximo = max(res.stand),
          SD = sd(res.stand),
          valorp_Shapiro = shapiro.test(res.stand)$p.value,
          valorp_Adarlin = ad.test(res.stand)$p.value,
          valorp_KS = ks.test(res.stand,"pnorm")$p.value,
          Asimetria = skewness(res.stand),
          Curtosis = kurtosis(res.stand),
          valorp_ncVtest = ncvTest(mod.cacrec)$p,
          valorp_bptest = bptest(mod.cacrec)$p.value) %>%
  data.frame() %>%
  t()

## Comparaciones multiples
HSD.test(y = Datos$CREC,
       trt = Datos$TRATA,
       DFerror = df.residual(mod.cacrec),
       MSerror = deviance(mod.cacrec)/df.residual(mod.cacrec),
       alpha = 0.05,
       group = FALSE)$comparison %>%
  mutate(CME = anova(mod.cacrec)["Residuals", "Mean Sq"],
       Cohen = difference / CME,
       across(where(is.numeric), ~ round(., 2))) %>%
  dplyr::select(difference, Cohen, pvalue)

## 4.3. Etapa de Engorde

# Resumen descriptivo
Datos %>%
```

```
  group_by(TRATA) %>%
  summarise(n = n(),
            Media = mean(ENG),
            Minimo = min(ENG),
            Maximo = max(ENG),
            SD = sd(ENG),
            CV = 100*SD/Media,
            MAD = median(abs(ENG-median(ENG))),
            Z = max(abs((ENG-Media)/SD))) %>%
  data.frame()

# Estimar el modelo
mod.caeng <- lm(ENG ~ TRATA, data = Datos)

# Resumen del modelo
summary(aov(mod.caeng))

# Coeficiente de variacion
SD <- sqrt(deviance(mod.caeng)/df.residual(mod.caeng))
100*SD/mean(predict(mod.caeng))

# Coeficiente de determinación
summary(mod.caeng)

# Analisis de los residuales estandarizados
data.frame(res.stand = rstandard(mod.caeng)) %>%
  summarise(Minimo = min(res.stand),
            Maximo = max(res.stand),
```

```
          SD = sd(res.stand),
          valorp_Shapiro = shapiro.test(res.stand)$p.value,
          valorp_Adarlin = ad.test(res.stand)$p.value,
          valorp_KS = ks.test(res.stand,"pnorm")$p.value,
          Asimetria = skewness(res.stand),
          Curtosis = kurtosis(res.stand),
          valorp_ncVtest = ncvTest(mod.caeng)$p,
          valorp_bptest = bptest(mod.caeng)$p.value) %>%
  data.frame() %>%
  t()

## Comparaciones multiples
HSD.test(y = Datos$ENG,
        trt = Datos$TRATA,
        DFerror = df.residual(mod.caeng),
        MSerror = deviance(mod.caeng)/df.residual(mod.caeng),
        alpha = 0.05,
        group = FALSE)$comparison %>%
  mutate(CME = anova(mod.caeng)["Residuals", "Mean Sq"],
        Cohen = difference / CME,
        across(where(is.numeric), ~ round(., 2))) %>%
  dplyr::select(difference, Cohen, pvalue)

## 4.4. Etapa Total

# Resumen descriptivo
Datos %>%
  group_by(TRATA) %>%
```

```
  summarise(n = n(),
            Mediana = median(TOT),
            Media = mean(TOT),
            Minimo = min(TOT),
            Maximo = max(TOT),
            SD = sd(TOT),
            CV = 100*SD/Media,
            MAD = median(abs(TOT-median(TOT))),
            Z = max(abs((TOT-Media)/SD))) %>%
  data.frame()

# Estimar el modelo
mod.catot <- lm(TOT ~ TRATA, data = Datos)

# Resumen del modelo
summary(aov(mod.catot))

# Coeficiente de variacion
SD <- sqrt(deviance(mod.catot)/df.residual(mod.catot))
100*SD/mean(predict(mod.catot))

# Coeficiente de determinación
summary(mod.catot)

# Analisis de los residuales estandarizados
data.frame(res.stand = rstandard(mod.catot)) %>%
  summarise(Minimo = min(res.stand),
            Maximo = max(res.stand),
```

```
          SD = sd(res.stand),
          valorp_Shapiro = shapiro.test(res.stand)$p.value,
          valorp_Adarlin = ad.test(res.stand)$p.value,
          valorp_KS = ks.test(res.stand,"pnorm")$p.value,
          Asimetria = skewness(res.stand),
          Curtosis = kurtosis(res.stand),
          valorp_ncVtest = ncvTest(mod.catot)$p,
          valorp_bptest = bptest(mod.catot)$p.value) %>%
  data.frame() %>%
  t()

## Comparaciones multiples

# SNK sin corrección
## Comparaciones multiples
HSD.test(y = Datos$TOT,
        trt = Datos$TRATA,
        DFerror = df.residual(mod.catot),
        MSerror = deviance(mod.catot)/df.residual(mod.catot),
        alpha = 0.05,
        group = FALSE)$comparison %>%
  mutate(CME = anova(mod.catot)["Residuals", "Mean Sq"],
        Cohen = difference / CME,
        across(where(is.numeric), ~ round(., 2))) %>%
  dplyr::select(difference, Cohen, pvalue)

# Prueba no parametrica de kruskall wallis
library(FSA)
```

```
kruskal.test(TOT ~ TRATA, data = Datos)

comp.krusk <- kruskal(y = Datos$TOT,

                      alpha = 0.05,

                      trt = Datos$TRATA,

                      p.adj = "none")

comp.krusk
```
